# Single-Cell Transcriptomic Census of Endothelial Changes Induced by Matrix Stiffness and the Association with Atherosclerosis

**DOI:** 10.1002/adfm.202203069

**Published:** 2022-09-12

**Authors:** Maedeh Zamani, Yu-Hao Cheng, Frank Charbonier, Vivek Kumar Gupta, Aaron T. Mayer, Alexandro E. Trevino, Thomas Quertermous, Ovijit Chaudhuri, Patrick Cahan, Ngan F. Huang

**Affiliations:** Department of Cardiothoracic Surgery, Stanford University, Stanford, CA 94305, USA; Stanford Cardiovascular Institute, Stanford University, Stanford, CA 94305, USA; Department of Molecular Biology and Genetics, Johns Hopkins University School of Medicine, Baltimore, MD 21205, USA; Institute for Cell Engineering, Johns Hopkins University School of Medicine, Baltimore, MD 21205, USA; Department of Mechanical Engineering, Stanford University, Stanford, CA 94305, USA; Department of Mechanical Engineering, Stanford University, Stanford, CA 94305, USA; Enable Medicine, Inc, Menlo Park 94025, USA; Enable Medicine, Inc, Menlo Park 94025, USA; Division of Cardiovascular Medicine, Stanford School of Medicine, Stanford, CA 94305, USA; Department of Mechanical Engineering, Stanford University, Stanford, CA 94305, USA; Department of Molecular Biology and Genetics, Johns Hopkins University School of Medicine, Baltimore, MD 21205, USA; Institute for Cell Engineering, Johns Hopkins University School of Medicine, Baltimore, MD 21205, USA; Department of Biomedical Engineering, Johns Hopkins University School of Medicine, Baltimore, MD 21205, USA; Department of Cardiothoracic Surgery, Stanford University, Stanford, CA 94305, USA; Stanford Cardiovascular Institute, Stanford University, Stanford, CA 94305, USA; Department of Chemical Engineering, Stanford University, Stanford, CA 94305, USA; Veterans Affairs Palo Alto Health Care System, Palo Alto, CA 94304, USA

**Keywords:** atherosclerosis, endothelial phenotypes, endothelial-to-mesenchymal transition, extracellular matrix stiffness, single-cell RNA sequencing

## Abstract

Vascular endothelial cell (EC) plasticity plays a critical role in the progression of atherosclerosis by giving rise to mesenchymal phenotypes in the plaque lesion. Despite the evidence for arterial stiffening as a major contributor to atherosclerosis, the complex interplay among atherogenic stimuli in vivo has hindered attempts to determine the effects of extracellular matrix (ECM) stiffness on endothelial-mesenchymal transition (EndMT). To study the regulatory effects of ECM stiffness on EndMT, an in vitro model is developed in which human coronary artery ECs are cultured on physiological or pathological stiffness substrates. Leveraging single-cell RNA sequencing, cell clusters with mesenchymal transcriptional features are identified to be more prevalent on pathological substrates than physiological substrates. Trajectory inference analyses reveal a novel mesenchymal-to-endothelial reverse transition, which is blocked by pathological stiffness substrates, in addition to the expected EndMT trajectory. ECs pushed to a mesenchymal character by pathological stiffness substrates are enriched in transcriptional signatures of atherosclerotic ECs from human and murine plaques. This study characterizes at single-cell resolution the transcriptional programs that underpin EC plasticity in both physiological or pathological milieus, and thus serves as a valuable resource for more precisely defining EndMT and the transcriptional programs contributing to atherosclerosis.

## 1. Introduction

Atherosclerosis, as defined by the progressive hardening and narrowing of the arteries, is a complex pathological condition that involves atherogenic activation and phenotypic modulation of vascular cell types, including endothelial cells (ECs), smooth muscle cells (SMCs), and inflammatory cells. In particular, ECs play a vital role in progression of atherosclerosis, by acquiring a pro-atherogenic phenotype in response to environmental stimuli including inflammation, oxidative stress, and vascular hemodynamics. Recent studies demonstrated that phenotypically modulated ECs are one of the prominent cell types within atherosclerotic lesions, and they are involved in the regulation of extracellular matrix (ECM) production, inflammatory cytokine secretion, and plaque stability.^[1,2]^ In vivo EC lineage-tracing studies in mice demonstrate that ≈45% of the fibroblast-like cells in atherosclerotic lesions are derived from ECs after 30 weeks of receiving a high fat diet,^[3]^ suggesting the importance of endothelial phenotypic change in response to extracellular microenvironmental cues in the progression of atherosclerosis. The plasticity of ECs in response to atherogenic stimuli gives rise to a cellular population with heterogeneous molecular and functional signatures.^[4]^ Endothelial-to-mesenchymal transition (EndMT) is a trans-differentiation process during which ECs gradually lose their endothelial identity, while acquiring mesenchymal phenotypic markers.^[5]^ EndMT accounts for the major EC phenotypic modulation responsible for the development of atherosclerosis, along with epigenetic regulation and less frequent endothelial transitions to hematopoietic stem cells and immune-like cells.^[6]^

Arterial stiffening is increasingly established as one of the predictive measures for cardiovascular diseases, including atherosclerosis. In particular, calcified human atherosclerotic plaque is reported to have a Young’s modulus of up to 0.7 GPa,^[7,8]^ which is strikingly higher compared to the biological stiffness of the endothelial basement membrane of ≈8 kPa.^[9]^ Despite the evidence for the association between atherosclerosis and stiffening of the arteries, the regulatory effect of ECM stiffness on cellular heterogeneity and phenotypic modulation of ECs, particularly EndMT, remains poorly defined, mainly due to the complex interplay among mechanical and molecular stimuli involved in progression of atherosclerosis. Previous studies demonstrate that hypertrophied rabbit hearts were associated with a stiffer myocardium, more frequent occurrence of EndMT, and more collagen deposition, as compared to healthy myocardium.^[10]^ However, the direct effect of stiffness on EndMT is difficult to study in multi-factorial pathological environments. Stiffness-regulated EndMT has been explored at the population level in in vitro studies using human umbilical vein ECs and valvular ECs cultured on pathologically stiff substrates.^[11,12]^ However, such in vitro systems based on global cell population analysis lack sufficient resolution to reveal the heterogeneity of the process. Moreover, the effect of matrix stiffness at the single-cell level is yet to be described in such an in vitro model.

Accordingly, the objective of this work is to apply single-cell RNA-sequencing (scRNA-seq) to explore the endothelial phenotypic modulation and cellular heterogeneity in response to substrate stiffness. Recent advances in droplet-based scRNA-seq now enable transcriptomic analysis of thousands of cells at the single-cell level.^[13–15]^ Using this technology, the transcriptional signatures of cellular subpopulations can be revealed, along with the degree of heterogeneity in the cell population. With the development of large-scale scRNA-seq techniques, emerging studies reveal the complex and dynamic nature of atherosclerotic plaque, as well as the contribution of multiple cell types at various pathophysiological activation states in the progression of atherosclerosis.^[6,16–19]^ Recently, scRNA-seq has revealed many insights into the role of SMCs in giving rise to mesenchymal-like lineages during the progression of atherosclerosis.^[16,18]^ However, little is known about the contribution of ECs to various mesenchymal-like lineages within atherosclerotic lesions. One report demonstrates endothelial heterogeneity in atherosclerotic plaque and the regulatory role of vascular microenvironment on endothelial plasticity.^[6]^ However, the effect of ECM biomechanics on EC phenotypic modulation and pathogenesis of atherosclerosis has not been explored at the single-cell level. Therefore, it remains unknown if ECM stiffness plays a role in endothelial heterogeneity by regulating cell trans-differentiation, atherogenic activation, epigenetic modification, and gene networks.

Here we show that endothelial phenotypic switches, including those resulting from EndMT, are regulated by substrate stiffness. We further show that the effect of transforming growth factor-*β* (TGF-*β*), a chemical inducer of EndMT, can be partially abrogated by matrix stiffness. We employ scRNA-seq as a powerful tool to identify the effect of stiffness on single-cell transcriptional reprogramming and heterogeneity of ECs in vitro. We further explore the dynamics of endothelial transitions directed by matrix stiffness and identify key transcription factors and target gene networks that selectively regulate the stiffness-modulated phenotypic changes. To reveal the translational relevance of these findings, we demonstrate that the subcluster of ECs enriched on the pathologically stiffness substrate closely resembles the transcriptional signature of ECs from clinical atherosclerotic lesions. This in vitro platform and scRNA-seq characterization serve as a valuable resource for studying EndMT and the genes that drive endothelial phenotypic changes under pathological stiffness at the single-cell level.

## 2. Results

### 2.1. Substrate Stiffness Modulates ECs Morphology and Phenotype

We first investigated the effects of substrate stiffness on primary human coronary ECs (HCAECs) cultured on soft polyacrylamide hydrogels of physiological stiffness (4 kPa), in comparison to cells cultured on stiff tissue culture (TC) plates that mimic pathological stiffness, both modified with fibronectin. As an ECM that supports EC adhesion,^[20–22]^ fibronectin is also reported to be one of the earliest ECM proteins deposited at sites of atherosclerosis-prone arteries, in addition to promoting atherosclerotic lesion formation.^[23,24]^ To confirm fibronectin’s bioactivity, we stained for fibronectin using immunofluorescence staining (Figure S1A, Supporting Information) and quantified the amount of fibronectin present on 4 kPa and TC to be 1084.5 ± 81.8 and 314.8 ± 57.3 ng cm^−2^, respectively, which were both much higher than the amount required to obtain maximum EC attachment and spreading (i.e., ≈80 ng cm^−2^).^[25]^ We also quantified cell attachment of both substrates modified with fibronectin and found no significant difference in cell attachment on 4 kPa and TC (Figure S1B, Supporting Information). These results suggest that both substrates provide sufficient ligands for cell attachment and likely provide a similar degree of fibronectin bioactivity.

**Figure 1.**
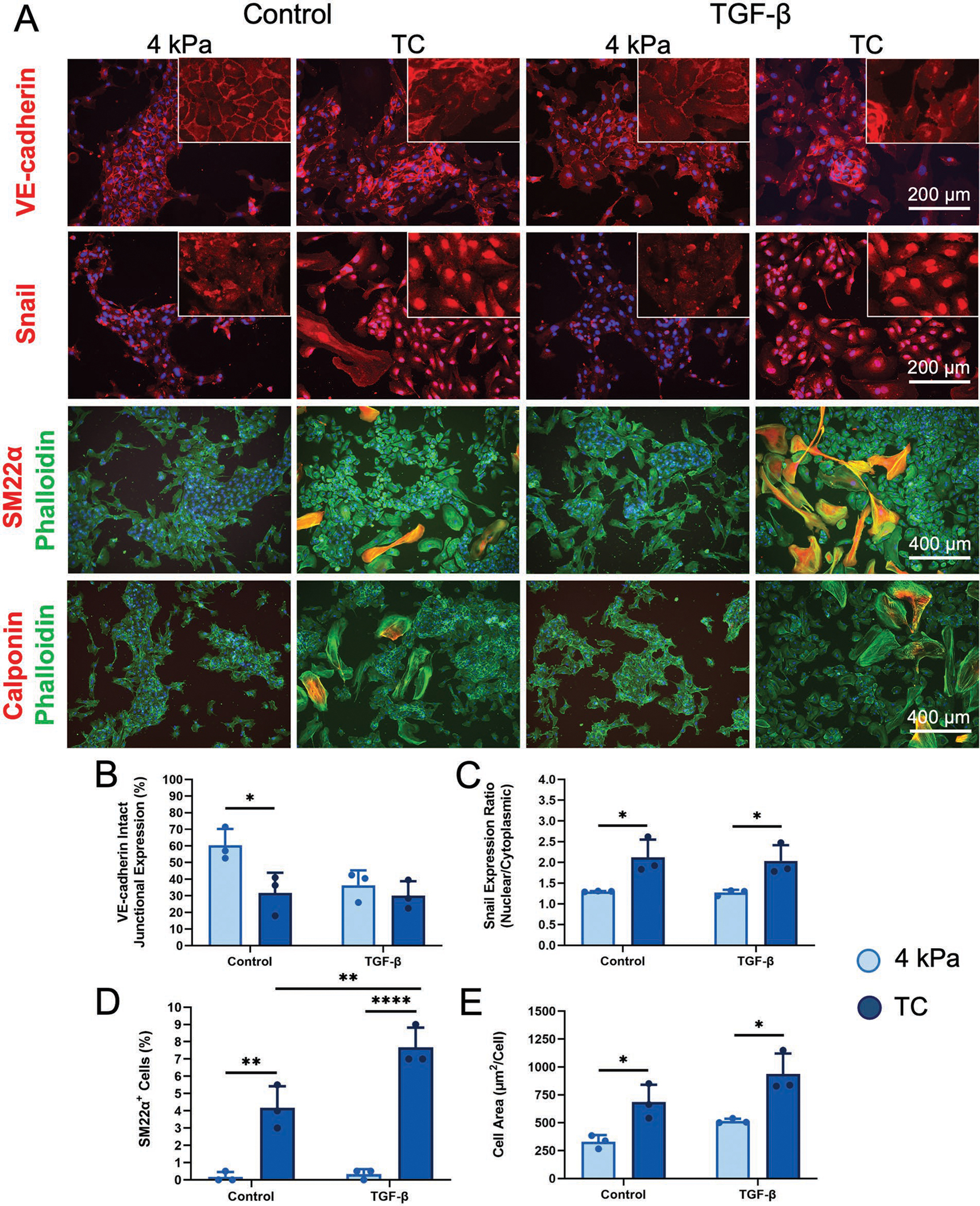
Substrate stiffness induces morphological and phenotypic changes in vascular endothelial cells (EC). A) Immunofluorescence staining of human coronary artery ECs cultured on physiological stiffness 4 kPa substrate or pathological stiffness tissue culture (TC) plastic, in the absence (Control) or presence of TGF-*β* stimulation after 14 days. Pathological stiffness TC substrate induced the loss of VE-cadherin junctional expression, the activation and translocation of snail-1 from cytoplasm to nucleus, the acquiring of mesenchymal markers SM22*α* and calponin, and cytoskeletal reorganization. Images were acquired at 20X for VE-cadherin and snail-1 and at 10X for SM22*α* and calponin. Scale bars represent 200 μm for VE-cadherin and snail-1, and 400 μm for SM22*α* and calponin. Quantitative analysis of B) VE-cadherin intact junctional expression; C) Snail-1 nuclear/cytoplasmic expression ratio; and D) Percentage of SM22*α* positive cells. E) Average cell area, based on fluorescent images. Data are shown as mean ± STD (*n* = 3). Data points represent average for each *n*. *p < 0.05, **p < 0.005, ****p < 0.00005.

After 14 days of ECs culture in endothelial basal media containing 5% fetal bovine serum, the cells were immunofluorescently stained for the endothelial phenotypic marker, VE-cadherin, a prominent cell adhesion molecule at endothelial junctions. Whereas cells on the 4 kPa substrate showed uniform junctional expression, in stark contrast, many ECs cultured on TC substrates showed a loss of the junctional expression of VE-cadherin (Figure 1A;Figure S2, Supporting Information). TGF-*β* induced the loss of VE-cadherin junctional expression to a lesser extent on both 4 kPa and TC substrates. Quantification of VE-cadherin expression demonstrated that the percentage of the cells with intact junctional expression on 4 kPa substrate was 60 ± 0.7%, which was significantly reduced to 32 ± 9% on the stiff substrate (*p < 0.05) (Figure 1B). TGF-*β* stimulation did not induce significant reduction in the cell-cell intact junctional expression on both 4 kPa and TC substrates.

With the marked loss of junctional expression in VE-cadherin on TC, we sought to investigate the potential of an endothelial phenotypic change by performing immunofluorescence staining of snail-1, one of the main markers associated with EndMT. We observed markedly different degrees of snail-1 activation based on its subcellular expression. In particular, snail-1 activation, as indicated by nuclear localization, was predominant in the cells cultured on the TC substrate, compared to cells cultured on 4 kPa (Figure 1A). Since snail-1 activity is highly regulated by its subcellular localization, these results confirm the association of substrate stiffness on snail-dependent endothelial phenotypic transition. Interestingly, the activation of snail-1 was primarily modulated by stiffness, as the translocation of snail-1 from cytoplasm to nucleolus was not observed for the cells stimulated with TGF-*β* on the 4 kPa substrate. To support these observations, snail-1 subcellular expression was quantified based on the nuclear/cytoplasmic expression ratio, in which a significantly increased ratio was found for the cells on TC (2.12±0.43) as compared to 4 kPa (1.30 ± 0.01) (*p < 0.05) (Figure 1C). Furthermore, cells in the presence of TGF-*β* stimulation showed similar findings of significantly higher nuclear/cytoplasmic ratio on TC (2.03 ± 0.38) as compared to 4 kPa (1.27 ± 0.07) (*p < 0.05).

Based on the finding of increased snail-1 translocation that suggested a phenotypic change towards mesenchymal phenotype, we further probed the protein expression of two mesenchymal/smooth muscle cell phenotypic markers, namely TAGLN (SM22*α*) and calponin. Both proteins were largely absent in 4 kPa substrates, whereas on TC substrates the expression of both SM22*α* and calponin were observed among cells that shared similar morphological characteristics of: 1) lacking cobblestone morphology, 2) having disrupted cell-cell junctions, and 3) having relatively larger cell area (Figure 1A). A similar finding was also observed among TGF-*β* induced cells, in which the cells cultured on 4 kPa were protected from a phenotypic change based on the scarcity of cells that expressed SM22*α* and calponin. The quantification of SM22*α*-expressing cells showed hardly any SM22*α*-expressing cells on 4 kPa substrate, regardless of TGF-*β* stimulation. In contrast, the SM22*α*-expressing cells on TC was 4.2% ± 1.3% of the total population, and it further increased to 7.7% ± 1.2% in the presence of TGF-*β* (**p < 0.005) (Figure 1D). These findings suggested that 4 kPa substrates preserved endothelial phenotype, while a stiffer TC substrate induced a mesenchymal phenotype in a subset of the cells, and that TGF-*β* induced phenotypic change could be inhibited on physiological 4 kPa substrates.

Besides phenotypic changes, we also explored the morphological changes of ECs in response to substrate stiffness by phalloidin staining of F-actin cytoskeletal assembly (Figure 1A). Morphologically, the cells on 4 kPa substrates formed multicellular clusters with tight cell–cell junction and exhibited the phenotypic cobblestone morphology of ECs with a uniform cell size distribution. The cobblestone morphology of ECs as well as their uniform size distribution on 4 kPa can also be observed more distinctly from VE-cadherin staining images at high magnification (Figure S2, Supporting Information). In the setting of increased stiffness, some of the ECs lacked cobblestone morphology, detached from multi-cellular clusters, and induced cytoskeletal reorganization with increased stress fiber assembly. With the increased intercellular gaps on TC substrates, some ECs exhibited greater cellular spreading, leading to increased cell size formation. In support of these observations, quantification of mean cell area of individual cells demonstrated a stiffness-mediated effect, in which the mean cell area was 2.1-fold larger TC (685 ± 156 μm^2^ per cell), compared to that on 4 kPa substrates (330 ± 60 μm^2^ per cell) (Figure 1E) (*p < 0.05). The TGF-*β*-treated samples similarly showed a 1.8-fold increase in cell area between cells on 4 kPa stiffness (514 ± 21 μm^2^ per cell) and TC (937 ± 183 μm^2^ per cell) substrates (*p < 0.05).

Concomitant with a larger cell size on the pathological stiffness TC substrates was the broader range of cell sizes (Figure S3A, Supporting Information) that suggested morphological heterogeneity. As shown in the histogram of the cell area distribution (Figure S3A, Supporting Information), the majority of cells was within 1000 μm^2^ in the cell area. However, on TC substrates, we observed a higher frequency of cells that were relatively larger in cell area, sometimes approaching 9000 μm^2^ in cell area. To further associate cell area with mesenchymal markers, we quantified the distribution of SM22-expressing and calponin-expressing cells with cell size. Our results showed that the expression of these mesenchymal markers was associated with cell sizes larger than 1000 μm^2^ and were more frequent on TC substrates (Figure S3B,C, Supporting Information). These data suggested that the ECs on stiff TC substrates were a heterogeneous cell population with morphologically larger cells and that expressed mesenchymal markers like SM22 and calponin. In contrast, the cells cultured on 4 kPa physiological stiffness substrates were relatively more uniform in morphology and had very low incidence of SM22 or calponin protein expression.

Cell cycle S-phase marker Ki67 staining also demonstrated that the transitioning cells that withdrew from the multi-cellular clusters were in a proliferative state, which is an indication of endothelial activation and mesenchymal phenotype acquisition. The proliferative cells consisted of cells with average cell area (<1000 μm^2^ per cell) or cells with relatively larger area (1100–4000 μm^2^ per cell). Although a trend of more Ki67-positive cells was observed on the stiff TC substrate, the effect was not found to be significant (Figure S4A–C, Supporting Information). Together, the findings from phenotypic and morphological analysis suggest that the ECs on stiff TC substrates consisted of a highly morphologically and phenotypically heterogeneous cell population that was characterized by the induction of mesenchymal markers. In stark contrast, ECs maintained on physiological 4 kPa substrates were more protected from a mesenchymal transition. TGF-*β* treatment also contributed to disruption of cell-cell contacts and cell spreading, independent of matrix stiffness.

### 2.2. Stiffness-Mediated Modulation of EC Phenotype at the Transcriptional Level

**Figure 2.**
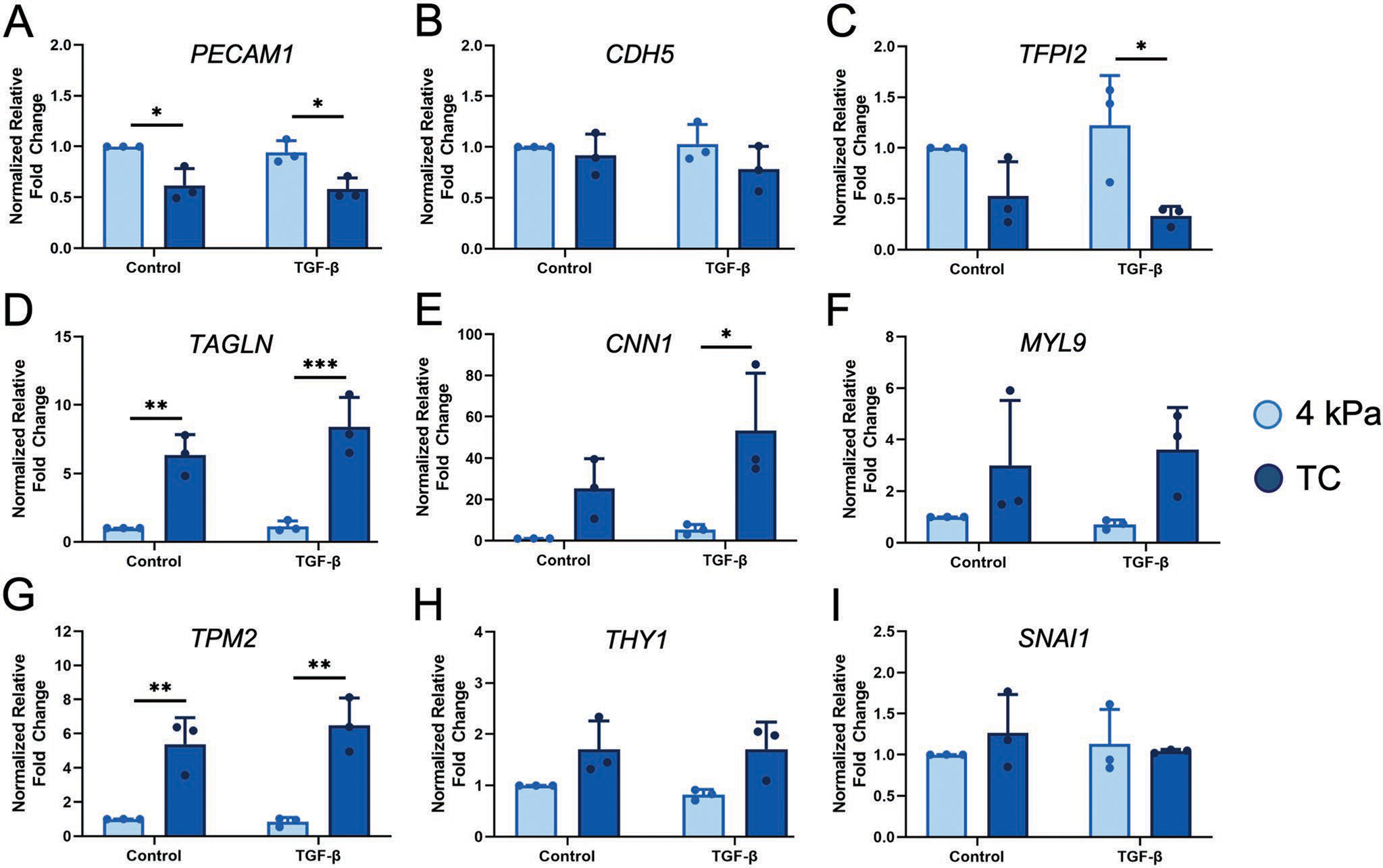
EC phenotypic modulation induced by matrix stiffness is validated by qPCR. Relative gene expression of A) PECAM1, B) CDH5, C) TFPI2, D) TAGLN, E) CNN1, F) MYL9, G) TPM2, H) THY1, and I) SNAI1 for human coronary artery ECs cultured on 4 kPa substrate or TC, either without (Control) and with TGF-*β* stimulation after 14 days. Gene expressions are normalized to *GAPDH* and are relative to 4 kPa as control. Data are shown as mean ± STD (*n* = 3). Data points represent average for each *n*. *p < 0.05, **p < 0.005, ***p < 0.0005.

To further characterize endothelial phenotypic transition at the transcriptional level, qPCR was performed on ECs cultured on 4 kPa hydrogel and TC, in the presence or absence of TGF-*β* stimulation. Our qPCR results demonstrate that substrate stiffness regulates the expression of endothelial markers *CD31* (*PECAM-1*), *VE-cadherin* (*CDH5*), and *TFPI2* after 14 days (Figure 2A–C). The relative fold-change gene expression of the cell adhesion marker, *PECAM-1*, was significantly downregulated to 0.62 ± 0.17 on TC (*p < 0.05) and 0.58 ± 0.11 (*p < 0.05) on TC with TGF-*β* stimulation, relative to the expression on 4 kPa substrates (1.00 ± 0.00, Figure 2A). Although transcriptional modulation of *CDH5* was not significantly different between substrates (Figure 2B), the expression of tissue factor pathway inhibitor-2 *(TFPI-2)*, an endothelial phenotypic marker associated with endothelial extracellular matrix integrity,^[26]^ was significantly downregulated on the stiff TC substrate in the presence of TGF-*β* (0.33 ± 0.09), in comparison to 4 kPa substrates (1.00 ± 0.00, *p < 0.05) (Figure 2C).

Concomitant with the downregulation of endothelial gene expression on stiff TC substrates was a significant upregulation in mesenchymal/smooth muscle cell markers, including *TAGLN*, calponin (*CNN1*), and myosin light chain-9 (*MYL9)* (Figure 2D–H). The relative fold expression of *TAGLN* was 6.36 ± 1.47-fold higher on TC substrates than on 4 kPa substrates (**p < 0.005). When cultured on TC substrates with TGF-*β* induction, the cells showed an 8.38 ± 2.17-fold higher expression of *TAGLN*, compared to on 4 kPa substrate with TGF-*β* stimulation (***p < 0.0005). These results are in accordance with observations for TAGLN and calponin protein expression (Figure 1A; Figure S3B,C, Supporting Information), indicating that the stiff substrate induces EndMT, whereas physiological 4 kPa substrate is favorable for maintaining ECs phenotype, even in the presence of TGF-*β* induction.

In contrast to results showing stiffness-mediated activation of snail-1 at the protein level, at the transcriptional level we did not observe significant stiffness-induced or TGF-*β*-activated differences in *SNAI1* expression after 14 days (Figure 2I) or intermediate time-point of 6 days (Figure S5I, Supporting Information). This finding suggests that *SNAI1* gene upregulation likely occurred at time points earlier than 6 days, since *SNAI1* regulates the early steps of EndMT.^[27]^ Nevertheless, we observed distinct post-transcriptional modification of snail-1 protein expression at subcellular level (Figure 1A). Additionally, transcriptional analysis at an intermediate time-point of 6 days demonstrated many similarities in gene expression patterns to that of day 14 (Figure S5, Supporting Information). The gene expression studies were also performed using an intermediate stiffness hydrogel of 100 kPa, and the results were compared to 4 kPa and TC (Figure S6, Supporting Information). Interestingly, after 14 days of HCAECs culture with and without TGF-*β* stimulation, the expression of the endothelial and mesenchymal markers on 100 kPa substrate was shown to be at an intermediate level compared to 4 kPa and TC, demonstrating the consistent effect of stiffness on phenotypic modulation of ECs.

Taken together, the protein and qPCR quantification of cellular phenotype and morphology demonstrates that ECs on the pathological stiffness TC substrates were relatively more heterogeneous than cells on physiologically stiffness 4 kPa substrates, while also gaining the expression of mesenchymal markers. Additionally, to a lesser extent, TGF-*β* also plays a role in promoting the expression of mesenchymal markers.

### 2.3. Single-Cell RNA Sequencing Reveals Three Major Transcriptional States

To uncover the transcriptional basis of stiffness-regulated mesenchymal activation and cellular heterogeneity on TC substrates, we performed scRNA-seq on human ECs cultured for 14 days on 4 kPa hydrogel or TC, in the presence or absence of TGF-*β* stimulation. After quality control filtering based on mitochondrial transcript content and total transcript counts, we obtained 21644 high-quality ECs (4781 cells from 4 kPa, 3699 cells from 4kPa+TGF-*β*, 6548 cells from TC, and 6616 cells from TC+TGF-*β*).

**Figure 3.**
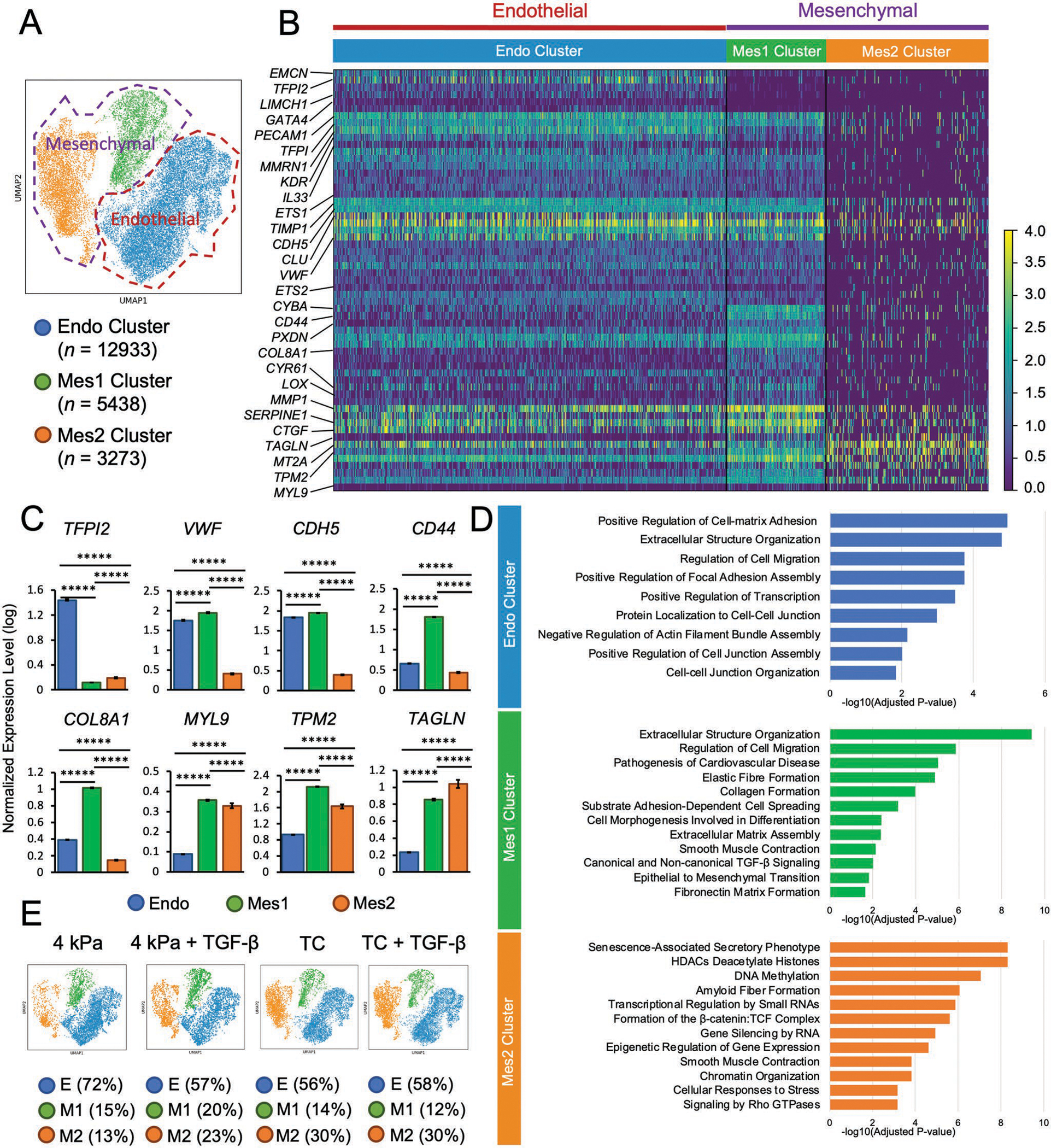
Single cell RNA Sequencing identifies three distinct cell clusters. A) Unsupervised clustering reveals three distinct EC clusters, namely Endo, Mes1, and Mes2. B) Heat map shows differences in differential gene expression among the clusters. Endo was enriched in typical EC markers (PECAM1, CDH5, and VWF) and functional genes to maintain healthy ECs (TFPI2, EMCN, and GATA4). Mes1 was marked by the expression of mesenchymal and ECM-related genes (LOX, MMP1, and COL8A1). Mes2 expressed multiple mesenchymal features (TAGLN, MYL9, and TPM2). C) Quantitative comparison of selected EC and Mesenchymal markers across clusters. Data shown as mean ± STD, *****p < 0.000005. D) Gene set enrichment analysis (GSEA) identified functional features of each distinct cluster. Endo showed signaling pathways related to cell-matrix adhesion and cell-cell junction organization. Mes1 gained multiple EndMT signatures, and Mes2 was enriched in both mesenchymal features and multiple signatures associated with active epigenetic regulation and chromatin organization. E) Stiffness-mediated cell distribution in the clusters. Pathological stiffness substrate and TGF-*β* decrease the percentage of Endo cells and increase the percentage of Mes cells.

To identify the major transcriptional states of ECs in these conditions, we pooled the datasets from four different conditions and performed unsupervised clustering, revealing three distinct clusters (Figure 3A). More than half of the cells were in the main cluster enriched in typical EC markers, such as *PECAM1, VWF, and KDR*, as well as the genes responsible for maintaining non-inflammatory/healthy ECs, including *TFPI2, EMCN*, and *GATA4*. Therefore, we defined the cluster as endothelial-like (Endo). Cells in this cluster simultaneously expressed high levels of some cell adhesion/activation-related genes (e.g., *MMRN1, LIMCH1, ETS2* and *IL-33*), as well as genes associated with inhibition of mesenchymal phenotype, including *MGP*, *TIMP1*, and *CLU*. These results suggest that this major cluster consists of ECs that are in an activated state. The remaining cells clustered in two distinct mesenchymallike (Mes) clusters that expressed varying degrees of markers associated with endothelial phenotypic modulation and mesenchymal markers. One cluster (Mes1) was marked by expression of ECM-related genes such as *LOX*, *MMP1, COL8A1*, and *PXDN*; mechanosensitive genes such as *CTGF*, *SERPINE1*, and *CYR61*; and cell adhesion molecules such as *CD44* and *CDH2*. Mes1 exhibited a blended expression phenotype with components of the mesenchymal (listed above) and endothelial genes (*VWF* and *CDH5*). Unlike Mes1, the Mes2 cluster consisted of the cells that did not express typical EC genes but rather expressed high levels of mesenchymal genes such as *TAGLN*, *MT2A*, *MYL9*, and *TPM2* (Figure 3B,C; Figure S7A, Supporting Information).

To discover the biological functions of these populations, we next performed gene set enrichment analysis (GSEA) (Figure 3D). The Endo was enriched in signaling pathways closely related to ECM organization and cell-matrix adhesion, along with signatures linked to EC identity, such as cell-cell junction organization and negative regulation of actin filament assembly. In contrast to the Endo cluster, Mes1 was enriched in EndMT signatures, including epithelial-to-mesenchymal transition, TGF-*β* signaling and matrix formation related to collagen and fibronectin, as well as smooth muscle contraction. Mes2 was enriched in mesenchymal signatures such as smooth muscle contraction and amyloid fiber formation and other mechanosensing related pathways such as activation of *β*-catenin/TCF complex and Rho GTPases signaling. Mes2 was also enriched in signatures associated with active epigenetic regulation and chromatin organization, including deacetylase activity, DNA methylation, and gene silencing, suggesting that Mes2 was in a distinct suppressed gene expression epigenetic state.

### 2.4. Substrate Stiffness and TGF-*β* Regulates EC Phenotypic Modulation

We then compared the cluster distribution among each treatment group to evaluate the impact of stiffness and TGF-*β* on EC phenotypic modulation (Figure 3E). Of the ECs cultured on the soft 4 kPa substrate, 72% were Endo cells. Either increased stiffness or TGF-*β* reduced this percentage of Endo cells to 56–58%. The fact that the combination of substrate stiffness and TGF-*β* did not further reduce this proportion of Endo cells initially suggested a common molecular pathway. However, the abundance of Mes1 and Mes2 cells did vary in response to stiffness or TGF-*β* stimulation. On the 4 kPa substrates, the abundance of the Mes1 population was 15%, which was similar to the population on TC (14%). The addition of TGF-*β* increased the Mes1 proportion on the 4 kPa substrates but did not markedly change the relative distribution of Mes1 on TC. In contrast, Mes2 showed a stiffness-dependent increase, in which the 13% abundance on 4 kPa increased to 30% on TC. The distribution of the cells in Mes2 increased from 13% to 23% on 4 kPa with TGF-*β* stimulation, while the cell proportion remained unchanged under TGF-*β* stimulation on the stiff substrate. The fraction of *TAGLN* expressing cells also increased on the stiff substrate or upon TGF-*β* stimulus (Figure S7B, Supporting Information). The percentage of all *TAGLN* expressing cells increased from 0.16% to 0.7% by increasing the stiffness. Under TGF-*β* stimulation, the fraction of TAGLN expressing cells increased to 0.3% on 4 kPa substrate and further increased to 2% on stiff substrate. The majority of *TAGLN* positive cells were contributed by Mes2, in which the percentage of *TAGLN* expression cells increased from 2.4% to 6.9% on stiff substrates with TGF-*β* stimulation. These findings suggested that high stiffness induced a greater proportion of cells in Mes2 and a higher fraction of *TAGLN* expression cells in this cluster.

Next, we performed differential gene expression analysis to better understand the impact of our culture conditions on transcriptional state. Regardless of cluster, endothelial features such as *PECAM1*, *TFPI2*, and *CDH5*, were downregulated in high stiffness and TGF-*β* conditions, while mesenchymal features, including *TAGLN*, *MYL9*, and *TPM2* were upregulated by these stimuli (Figure S7C, Supporting Information). These results were consistent with differential gene expression analysis performed on a cluster-specific basis: low stiffness promoted EC features and high stiffness or TGF-*β* stimulation induced the expression of mesenchymal markers. In contrast to Mes1 and Mes2, the endothelial-like cells in Endo unexpectedly expressed higher levels of *CDH5* on the stiff substrate, probably due to *CDH5* mechanotransduction.^[28]^ Additionally, we compared the differential gene expression of integrin family of subunits, actin, paxillin, vinculin, focal adhesion kinase (*PTK2*), and talin on 4 kPa and TC, with and without TGF-*β* stimulation (Figure S7D, Supporting Information). The average expression of the cell adhesion and cytoskeletal molecules were comparable across four different conditions, suggesting the comparable anchoring ability of ECs on 4 kPa and TC. These results suggest that the differences in EC phenotypic modulation are primarily stiffness mediated.

To understand whether ECs on both soft and stiff substrate are biologically active, we reconstructed the gene regulatory networks by using context likelihood of relatedness (CLR) for each condition. We identified a consistent network that involved multiple critical transcription factors regulating endothelial and mesenchymal features across four different conditions, named common edges across all samples (Figure S8, Supporting Information). The network involved several EC regulators that were positively correlated to EC transcriptional features such as *CDH5*, *VWF*, *PECAM1*, *TIE1*, and negatively correlated to mesenchymal signatures including *TPM2*, *TAGLN*, and *MYL9*. The transcription factors governing the ECs features included the known EC regulators like *GATA2*, *FLI1*, and *HEY1*. *GATA2* governed several mediators related to EC functions, including *ENG* and *VCAM*. *FLI1* regulated *CDH5* and *PECAM1* expression in EC, which were critical to the development of the basement membrane. The common regulatory network suggests that the cells on both 4 kPa and TC substrates were biologically active and shared some molecular mechanisms in governing endothelial and mesenchymal features. To evaluate the impact of stiffness specifically on the network governing endothelial and mesenchymal features, we next identified the differential network that was only observed for either soft or stiff substrate. Interestingly, there were a significant number of edges that were present only on TC. The negative regulation of mesenchymal genes including *TPM2*, *TAGLN*, and *MYL9* by the known EC transcription factors such as *FLI1*, *ERG*, and *SOX18* was only observed on the stiff substrate. Additional genes, including *TWIST2* and *NFIB* contributed to regulate mesenchymal features, while most of the differential edges involved in regulating the endothelial features. These gene regulatory networks suggest that while endothelial cells were biologically active on both soft and stiff substrate, several networks were explicitly regulated on stiff substrate.

To investigate the roles of mechanotransduction on phenotypic modulation of ECs and transition between clusters, we evaluated the correlation between the known mechanotransduction elements^[29]^ and the common network reconstructed using the CLR method. The cluster-specific gene regulatory networks revealed a high degree of connectivity between endothelial and mesenchymal features and their regulators to the mechanotransduction elements in Endo, which was gradually decreased by transition to Mes1 and Mes2 (Figure S9, Supporting Information). These results suggest a significant role of mechanotransduction in phenotypic modulation of ECs in Endo, an effect that is less pronounced for the cells going through phenotypic modulation in Mes1 and Mes2.

### 2.5. EC Sub-States are Differentially Impacted by Substrate Stiffness and TGF-*β*

**Figure 4.**
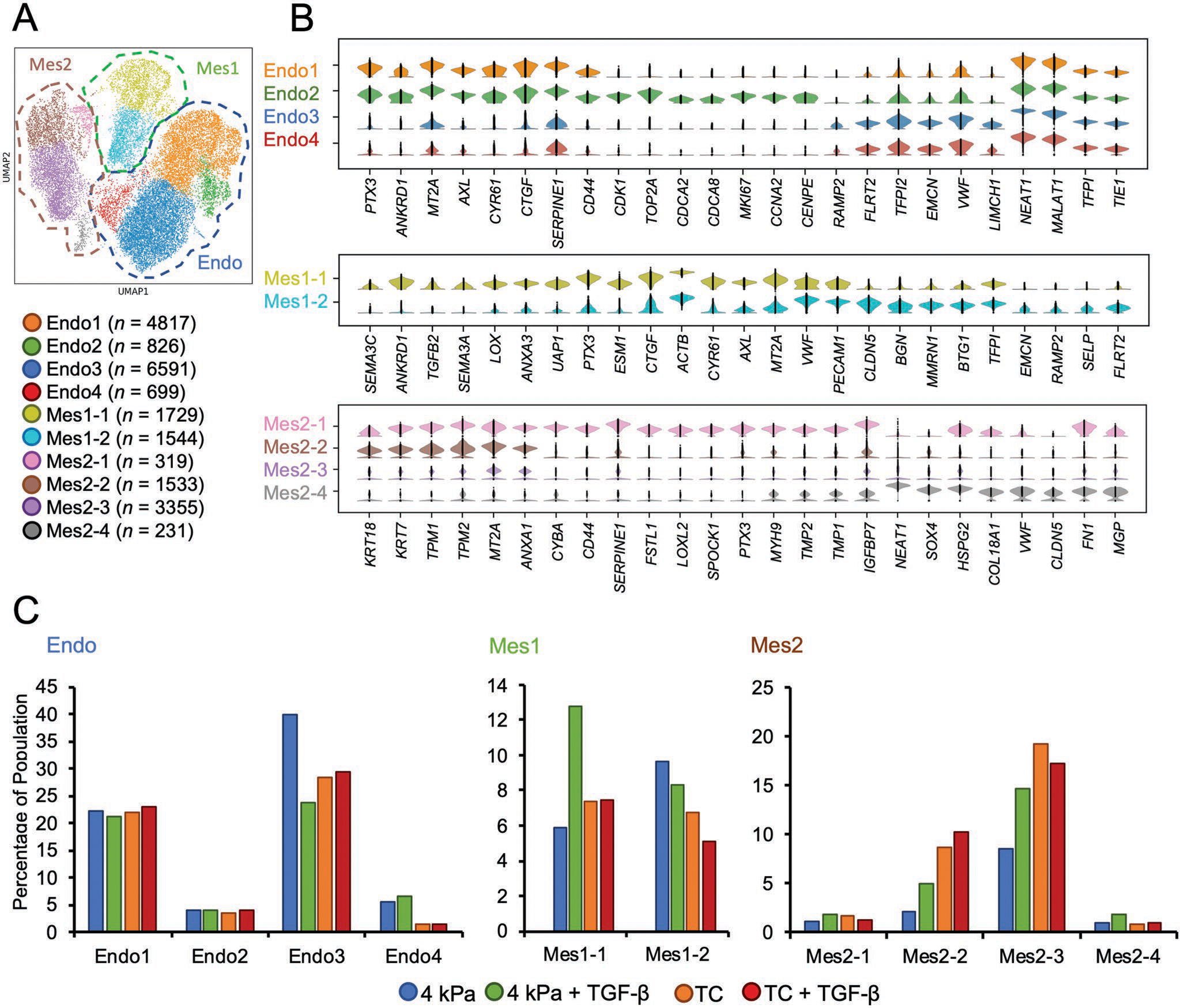
Cluster dynamics are revealed by sub-cluster analysis. A) Sub-clustering analysis revealed four distinct subclusters in Endo cluster, two in Mes1 and four in Mes2. B) The differential genes analysis revealed a consistent Endo-to-Mes gradient pattern within each cluster. Endo1 and Endo2 substates expressed more ECM and EndMT related genes than Endo3 and Endo4. Mes1–1 had more prominent mesenchymal features compared to Mes1–2, and Mes2–1 and Mes2–2 expressed a higher degree of mesenchymal features than Mes2–3 and Mes2–4. C) The distribution analysis across treatment conditions revealed the subclusters highly impacted by the stiffness or TGF-*β* induction, including Endo4, Mes2–2, and Mes2–3.

To uncover the heterogeneity of each distinct transcriptomic state, we performed the sub-clustering analysis for each primary cluster, revealing four distinct subclusters in the Endo, two in Mes1, and four in Mes2 (Figure 4A). The differentially expressed genes revealed a gradient of endothelial and mesenchymal signatures within Endo, Mes1, and Mes2 (Figure 4B). Several sub-states, including Endo1, Endo2, Mes1–1, Mes2–1, and Mes2–2, expressed more ECM and EndMT related genes, including *PTX3*, *AXL* or *TPM2* and also mechanosensitive transcripts such as *CYR61* and *CTGF*. Meanwhile, the remaining sub-states expressed markers of physiologically normal endothelium, such as *EMCN*, *VWF*, or *FLRT2*. We did not detect any genes that were preferentially expressed in Mes2–3, consistent with a broadly repressive epigenetic state. These intra-cluster heterogeneities suggest dynamic states within each cluster and the possible routes of transitions among endothelial and mesenchymal states.

To assess the impact of stiffness and TGF-*β* on EC phenotypic modulation, we next compared the sub-cluster distribution across treatments. The subclusters with more EC character, including Endo3, Endo4, and Mes1–2, had a substantially higher fraction at physiological 4 kPa stiffness compared to TC, or upon TGF-*β* stimulation (Figure 4C). Endo3 consisted of 49.93% of the cell population on 4 kPa, decreasing to 23.66% upon TGF-*β* induction or 28.36% on TC. Endo4 was also primarily composed of the cells cultured in physiological stiffness conditions (6–7% on 4 kPa, which dropped to 1% on the stiff substrate). In contrast, the subclusters with more mesenchymal features, including Endo1, Endo2, Mes1–1, Mes2–1, and Mes2–2, were more populated on TC. The fraction of the cells in Mes1–1 increased with TGF-*β* induction only on low stiffness (from 5.88% to 12.76%) but not on TC (from 7.36% to 7.47%). With increasing stiffness, the fraction of Mes2–2 increased from 2% (4 kPa) to 9% (TC) in the absence of TGF-*β* induction, with corresponding values of 5% (4 kPa) to 10% (TC) upon TGF-*β* induction. Mes2–3 also shared a common pattern as Mes2–2, in which increasing the substrate stiffness doubled the subcluster size (from 8% to 19%), and the addition of TGF-*β* led to a further increase in 4 kPa (14%) but remained at the same level in TC (17%). These findings illustrate the interplay between substrate stiffness and TGF-*β*, suggesting their similar degree of regulatory effect on sub-state cell populations and cellular phenotypic modulation.

### 2.6. Trajectory Inference Predicts Complex Patterns of EC State Transition

**Figure 5.**
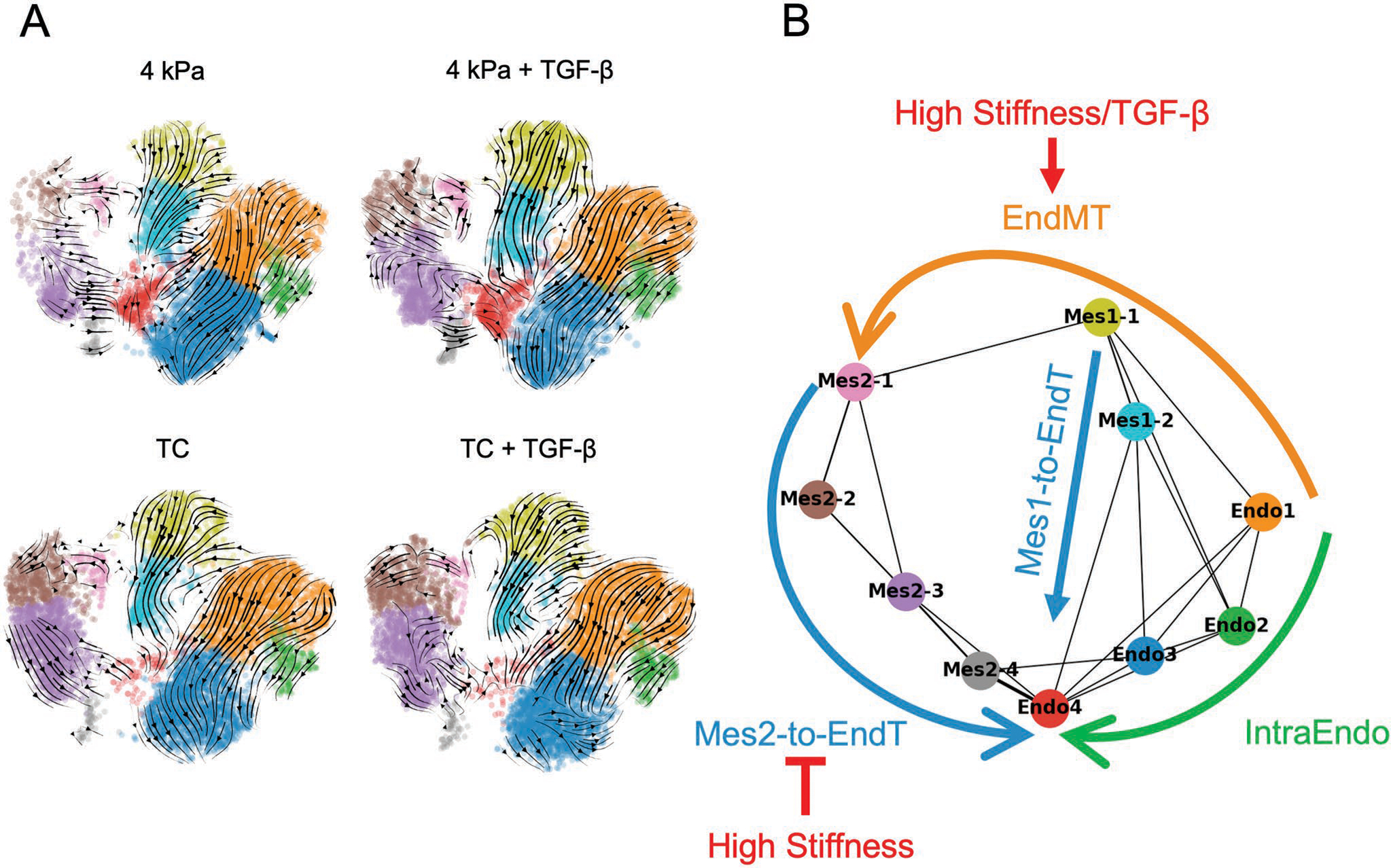
Trajectory Inference revealed complex circulatory dynamics. A) RNA velocity identified four distinct routes of EC state transitions, which included intraEndo, EndMT, Mes1-to-EndT, and Mes2-to-EndT. B) The non-directional connectivity evaluation using PAGA revealed comparable trajectories discovered by the RNA velocity.

To explore the potential fate relationships among the sub-clusters, we performed RNA velocity analysis, which predicts the future transcriptional state of cells by modeling transcriptional dynamics using the ratio of the unspliced to spliced transcript abundances.^[30]^ Overall, the inferred trajectories were similar across culture conditions. Cells on both 4 kPa and TC substrates exhibited a consistent flow within Endo, starting from the more mesenchymal like subclusters Endo1 and Endo2 and terminating in the more endothelial subcluster Endo3 (a trajectory that we refer to as “intraEndo”), regardless of TGF-*β* induction (Figure 5A). This analysis also predicted an endothelial to mesenchymal (EndMT) transition from Endo1 to Mes1–1 and Mes1–2, a transition more apparent in high stiffness conditions. Finally, we observed two unanticipated transitions from the mesenchymal to the endothelial states, which we refer to as mesenchymal-to-endothelial transitions (Mes-to-EndT). The first such transition, Mes-to-EndT1, in which cells transitioned from Mes1–1 to Mes1–2 to Endo4, occurred in all conditions. On the other hand, the second such transition, Mes-to-EndT2, in which cells transitioned from Mes2–3 to Endo4, was most strikingly apparent under physiological stiffness. This result would be consistent with a model in which the absence of an Endo4 state in the stiff condition samples is due, at least in part, to reduced number of mesenchymal cells transitioning back to an endothelial state except under physiological stiffness. The non-directional connectivity analysis of PAGA supports the putative trajectories discovered by the RNA velocity (Figure 5B).

### 2.7. Fluorescent In Situ Hybridization Reveals Unique Spatial Interaction Across Clusters

**Figure 6.**
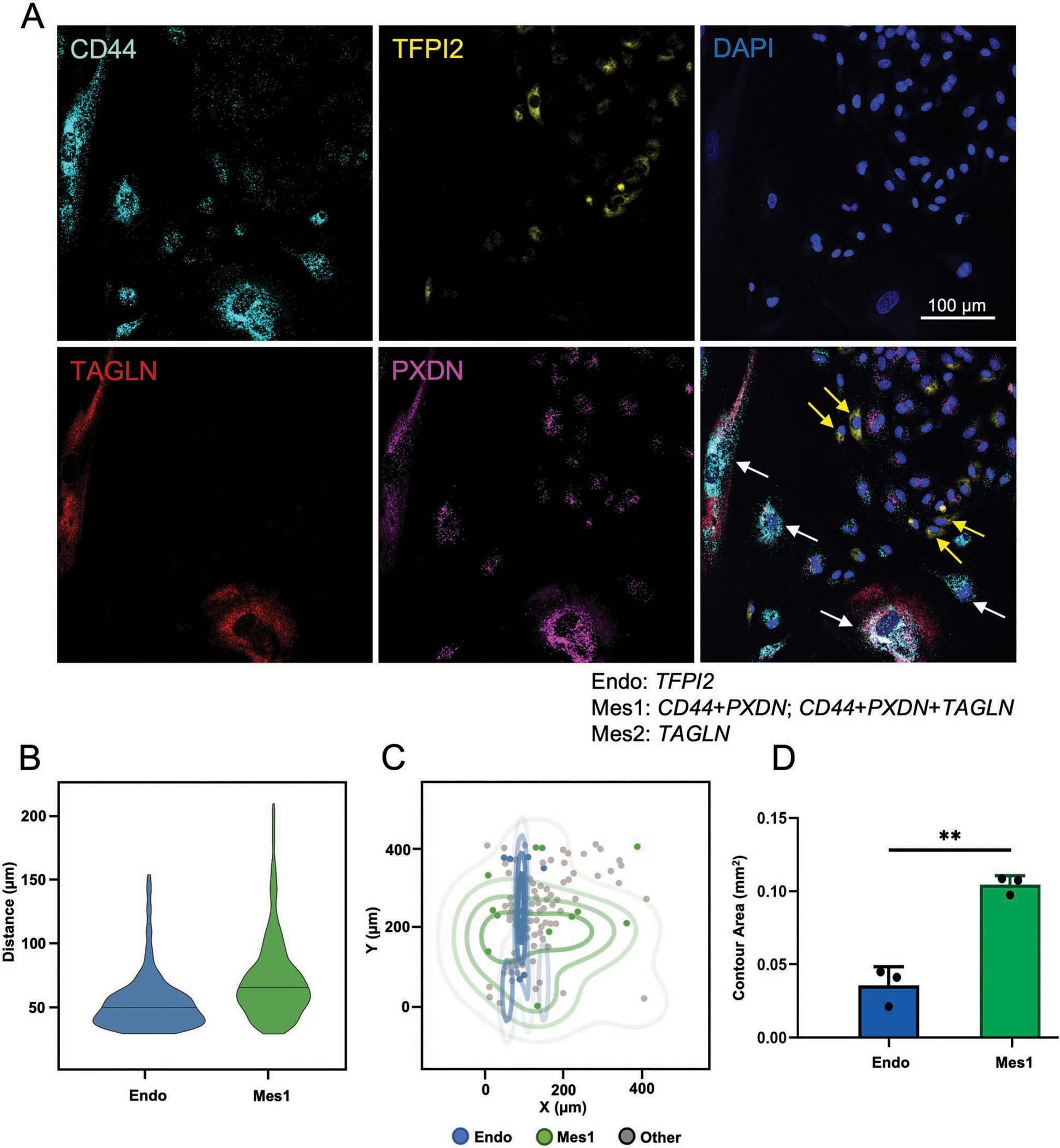
Spatial distribution of cell populations was demonstrated by fluorescent in situ hybridization (RNAscope). A) Confocal images of single RNA molecules probed by RNAscope for HCAECs cultured on TC substrate with TGF-*β* induction for 14 days. Endo cells were identified based on enrichment of TFPI2, and Mes1 cells were detected by co-expression of CD44+PXDN; or tri-expression of CD44+PXDN+TAGLN. TAGLN expressing cells (Mes2) could not be detected by RNAscope probes. Yellow and white arrows point to representative Endo and Mes1 cells, respectively. Scale bar represents 100 μm. B) Intra-cluster analysis of cell-cell distances computed for five nearest neighbors of each cell revealed a wider distribution for Mes1 cells compared to Endo. C) Quartiles of the spatial cell density distribution for endo (blue) and Mes1 (green) cells illustrated wider contours area for Mes1 cells compared to Endo, with Mes1 contours being circumferential to the ones for Endo cells D) Average area of 75% cell density contours for Endo and Mes1 cells demonstrated the wider spatial distribution of Mes1 compared to Endo cells. Data are shown as mean ± STD (*n* = 3). Data points represent average for each *n*. **p < 0.005.

In order to validate the sc-RNAseq results and to provide spatial analysis of cellular clusters, we performed in situ hybridization using RNAscope (Advanced Cell Diagnostics) for the cells cultured on TC with TGF-*β* stimulation for 14 days. Based on the sc-RNAseq results we probed for *TFPI2*, a highly expressed specific gene for Endo to locate the cells assigned to this cluster. The co-expression of *PXDN* and *CD44* was used to specifically identify the cells belonging to Mes1, since the cells in Endo and Mes2 negligibly co-expressed these two probes. *TAGLN* alone was abundantly expressed by the cells in both Mes1 and Mes2, but its tri-expression with *PXDN* and *CD44* was limited to the cells in Mes1. Figure 6A demonstrates that the majority of Endo cells targeted with *TFPI2* probe had small cell area, along with smaller distance among one another, and tended to form multicellular clusters in the culture.

The cells co-expressing *PXDN* and *CD44* that were associated with Mes1 showed a distinctively different spatial distribution pattern. These relatively larger cells were mostly circumferential to Endo cells, with a more random distribution in the culture. Unfortunately, the Mes2 cells could not be identified using RNAscope target probe *TAGLN*, probably due to overall lower RNA content of these cells compared with Endo1 and Mes1 clusters, as confirmed by sc-RNAseq (Figure S10A, Supporting Information).

Quantifying the RNAscope staining images, a total of 24.5% and 14.99% of the cells could be assigned to Endo and Mes1, respectively (Figure S10B, Supporting Information). Cells with detectable *PXDN* transcript only that could be associated with either Endo or Mes1 accounted for 13.53% of the total cell population. *CD44* transcription only was also probed in additional 17.92% of the cells, the ones that could potentially belong to any of the three clusters. The four target probes combination used for RNAscope was not detected in 29.07% of the cells in the culture. In order to analyze the spatial distribution of different cell populations in RNAscope images, we first explored the distances between the immediate neighbor cells from the same cluster. The Endo cells showed to have relatively smaller distribution of distances compared to Mes1 (Figure 6B), confirming the clustered spatial organization of Endo cells compared to more random distribution of Mes1 cells in the culture. We also visualized the spread of the cells and computed the quartiles of the cell density distribution for endo and Mes1 cells (Figure 6C). The results revealed wider contour areas for Mes1 cells compared to Endo, with Mes1 contours being circumferential to the ones for Endo cells. The average area of 75% density contour for Mes1 cells was 0.104 ± 0.006, which was significantly higher than the area of the spread for Endo cells (0.036 ± 0.012, **p < 0.005) (Figure 6D).

As an additional confirmation for the spatial organization of the cell populations, we used statistics 2D/3D Fiji plugin developed by Andrey et al.^[31]^ Based on their method of calculation explained in the methods section, the G-function value can be changed between 0 and 1. The high values of G-function indicate uniformity of the patterns, while low values are associated with clustered patterns. The analysis of the RNAscope images using this function resulted in G value of 0.01 ± 0.01 for Endo cells in the images, confirming the clustered distribution of Endo cells in the images. On the other hand, Mes1 cells analysis yielded G value of 0.88±0.15, indicating the more random distribution of Mes1 cells in the culture (**p < 0.005) (Figure S10C, Supporting Information). These results confirm the findings (Figure 6D) of a more clustered distribution of Endo cells, in contrast to Mes1 cells. These results are in line with immunofluorescence staining findings at protein level, where small area cells with intact cell-cell junction (Endo) tended to form multi-cellular clusters in the culture, and the large area cells that appeared detached from the multi-cellular clusters (Mes1) stained for the mesenchymal markers (Figure 1). Together, this data suggests that Endo and Mes1 cells have distinct spatial organization.

### 2.8. The Clinical Significance of Stiffness-Induced EC Phenotypic Change in Atherosclerosis

**Figure 7.**
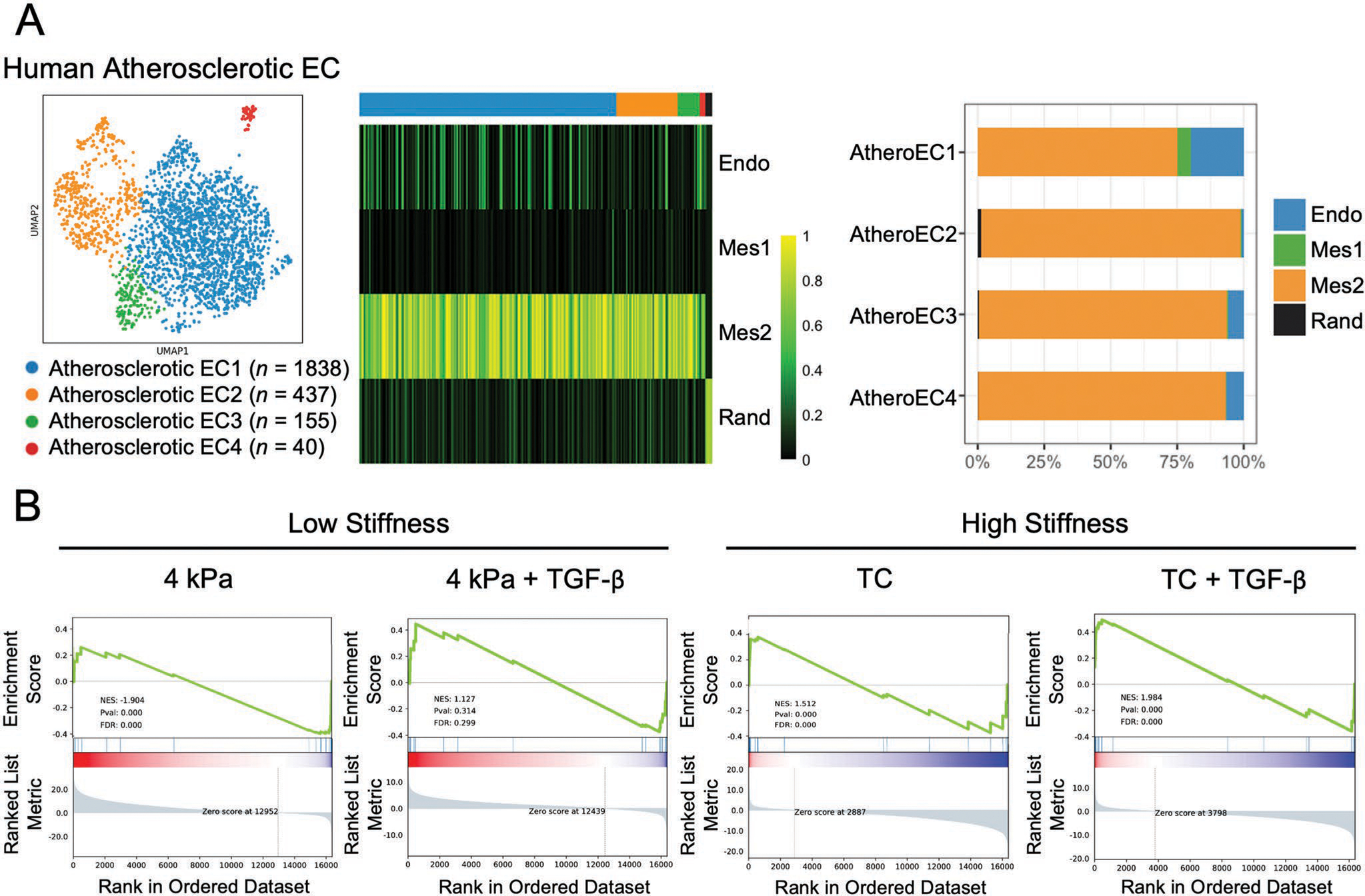
Associations of in vitro stiffness effects on ECs with clinical atherosclerotic lesions. A) The single-cell classification mapped most of the atherosclerotic ECs from human coronary artery atherosclerotic lesions as Mes2. B) Gene set enrichment analysis using pre-established gene list of signatures derived from atherosclerotic lesions showed a negative enrichment of the atherosclerotic features on physiological stiffness (4 kPa), while the stiff substrate promoted the pathological phenotype of ECs demonstrated by positive enrichment score.

To evaluate how the stiffness-mediated phenotypic changes in ECs correlate with atherosclerosis, we first compared the transcriptional signature of ECs from scRNA-seq of human coronary artery atherosclerotic lesions^[19]^ with our in vitro data. Using EC phenotypic markers (i.e., *PECAM1*, *CDH5*), we identified 2470 high-quality atherosclerotic ECs (atheroECs) from human atherosclerotic arterial samples, which could be further clustered into four distinct clusters. We applied the outperformed single-cell classification tool SingleCellNet to quantitatively assess how the atherosclerotic EC collected from the patient map to the clusters we identified. Notably, only 23% of AtheroEC1 were classified as our Endo cluster, and most of the atherosclerotic ECs were classified as Mes2 (Figure 7A), suggesting that the transcriptomic features of human atherosclerotic ECs are mostly similar to the ones of transitioning cells in this cluster. This suggests that the stiffness-mediated phenotypic modulation of ECs in Mes2 might contribute to pathogenesis of atherosclerosis.

As an additional validation, we compared our in vitro findings with scRNA-seq datasets derived from murine atherosclerotic brachiocephalic arteries and healthy ECs, taking advantage of endothelial lineage tracing capacity.^[16]^ The clustering of murine ECs formed four clusters, of which three were populated with atherosclerotic ECs and one cluster that was formed by healthy ECs (Figure S11, Supporting Information). The comparison of the gene signatures of these clusters with our in vitro subclusters demonstrated that the highest fraction of the cells classified as healthy ECs resided in subclusters Endo3 and Endo4 (the bona fide EC subclusters) and Mes1–2 and Mes2–4 (transitioning cells that regained their EC signatures). The remaining cells from activated Endo subclusters and Mes1 and Mes2 substates were predominantly assigned to murine AtheroEC2 cluster.

These findings confirmed that while the transcriptomic features of our substates with more endothelial signatures are partially similar to the ones from murine healthy ECs, the stiffness-mediated phenotypic signatures of our mesenchymal substates are highly similar to EC-derived cells in atherosclerotic lesions.

To further assess the extent to which cultured ECs mimicked the atherosclerotic ECs transcriptomic signatures, we performed gene set enrichment analysis using pre-established databases. We compiled a gene list of signatures derived from atherosclerotic lesion samples and literatures related to EndMT, and subsequently performed customized GSEA (Table S1, Supporting Information). The physiological stiffness showed a negative enrichment of the atherosclerotic features, while the difference became insignificant with TGF-*β* induction, supporting the hypothesis that physiological stiffness protects cells from acquiring pathological features associated with atherosclerosis. However, regardless of TGF-*β* induction, the stiff substrate promoted pathological phenotype of ECs, as demonstrated by positive enrichment of the atherosclerosis signatures (Figure 7B).

## 3. Discussion

Atherosclerotic lesions consist of multiple cell types derived from vascular cells undergoing phenotypic modulation in response to atherogenic stimuli, forming a highly complex and dynamic microenvironment. The strong evidence for arterial stiffness being one of the underlying factors contributing to development of atherosclerosis had led to clinical interventions aimed to restore the physiological state. These strategies rely on small molecules that interfere with ECM stiffening pathways involved with metalloproteinase activities, collagen crosslinking, and advanced glycation end products (AGEs).^[32–34]^ Our findings from endothelial and mesenchymal marker protein expression suggested that the ECs on pathological stiffness TC substrates consisted of a highly morphologically and phenotypically heterogeneous cell population that was characterized by the induction of mesenchymal markers. In stark contrast, ECs were more protected from the mesenchymal transition on physiological 4 kPa substrates. While transcriptomic and proteomic studies using in vivo samples with augmented arterial stiffness would better mimic the complex pathological conditions associated with atherosclerosis, the interpretation of the stiffness effect on pro-atherogenic modulation of individual cell types would be highly challenging due to the interplay among various cellular, biomolecular, and biomechanical components of atherosclerosis in the in vivo and clinical setting. Moreover, in the context of scRNA-seq, tissue dissociation and single cell isolation appeared to be critically important factors in regulation of gene expression and can be a source of heterogeneity in transcriptomic profile of the cells. Taking advantage of scRNAseq capability that allows the comparison between cell populations obtained using different techniques and species, our in vitro studies enabled us to independently investigate the differential effects of stiffness on EC heterogeneity and phenotypic modulation, followed by exploring the translational relevance of these findings in human and mouse atherosclerotic lesions.

With development of large-scale scRNA-seq techniques, recent studies investigated the role of SMCs in giving rise to mesenchymal-like lineages during the progression of atherosclerosis.^[16,18,19]^ ScRNA-seq of advanced brachiocephalic artery lesions from SMC lineage tracing mice revealed seven distinct SMC derived clusters in the lesions. The phenotypic modulation of SMC derived cells showed to be very dynamic, where at least three different cell subpopulations with osteogenic, proinflammatory, and ECM remodeling transcriptomic signature were derived from a transitional state of the cells highly expressed *lgals3*. Most of these SMC derived subpopulations from mice model were also shown to be present in atherosclerotic lesions of human coronary arteries.^[16]^ The SMC derived cells in murine and human atherosclerotic lesions were also found to trans-differentiate into fibroblast-like cells.^[19]^

While single cell transcriptional profiling has revealed new knowledge of SMCs phenotypic change during atherosclerosis, little is known about the cellular heterogeneity in the endothelial derived subpopulations in atherosclerotic lesions. The only study was published by Andueza et al. where they developed a mouse partial carotid ligation model, to study the effect of disturbed flow on ECs reprogramming. Their results revealed 8 clusters of ECs with high transcriptional heterogeneity and concluded that disturbed flow induces phenotypic modulation of ECs from atheroprotective to proatherogenic phenotype, including EndMT and endothelial to immune-like cell transition.^[6]^ Our scRNA-seq experiment is the first study focused on the effect of biomechanics of ECM on ECs phenotypic modulation and pathogenesis of atherosclerosis at the single cell level. We demonstrated that the matrix stiffness plays a determining role in endothelial heterogeneity through regulating ECs atherogenic activation, trans-differentiation to mesenchymal lineage and epigenetic modification.

We found three transcriptionally distinct clusters consistently shown in all different cell culture conditions, demonstrating that phenotypic modulation of ECs on substrates of varying stiffness and TGF-*β* induction did not involve developing a new distinct cell state or cell type. Although this observation may imply the potential of ECs undergoing phenotypic modulation even at physiological stiffness without TGF-*β* induction, we cannot exclude the possibility that our in vitro culture condition using growth factor-free media accelerated the induction of phenotypic modulation. For instance, the inhibitory role of fibroblast growth factor (FGF) and vascular endothelial growth factor (VEGF) signaling on TGF-*β* signaling and EndMT progression has been previously confirmed.^[1,10,35]^ Our gene expression analysis of the cell adhesion molecules also confirmed the comparable interaction of ECs with both substrates of varying stiffness, suggesting the stiffness being the main underlying regulator of ECs phenotypic modulation. This along with the formation of actin stress fibers across all culture conditions demonstrates that ECs were able to sense and respond to stiffness on both substrates.

The Mes clusters we identified showed very distinct features. We could observe a comparable expression of typical EC markers in Mes1 compared to Endo, which suggested that Mes1 was an intermediate state derived from Endo. The Mes2, unlike Mes1, showed unique features of downregulation of multiple EC and Mes1 features, and upregulation of some of the SMC markers. Based on the GSEA analysis, the global downregulation of genes in Mes2 might be driven by the epigenetic regulation that was involved in the conversion between Mes1 and Mes2. This is in line with previous studies demonstrating the stimulatory effect of ECM stiffness on epigenetic modification of epithelial cells,^[36]^ and the role of epigenetics in progression of EndMT.^[35,37]^ Our findings reveal intriguing evidence for ECs phenotypic modulation at both transcriptional and epigenomic level regulated by stiffness. This needs to be further assessed through integrated analysis of scRNAseq and scATAC-seq to simultaneously study these complementary aspects of gene regulation.

The increase in the mesenchymal-like subpopulations in response to stiffness or TGF-*β* induction was not only confirmed among three main clusters, but also at subcluster level. When we compared the same cluster among different culture conditions, we could also observe a significant decrease in endothelial features and increase in the mesenchymal signatures on stiff substrate and under TGF-*β* stimulation. One exception was *CDH5*, which was expressed at a higher level by endothelial-like cells in Endo on the stiff substrate, probably due to *CDH5* mechanotransduction.^[28]^ This finding was supported by immunofluorescence staining of VE-cadherin, in which ECs with intact cell–cell junctions located specifically within multicellular clusters expressed higher levels of junctional *CDH5* protein (VE-cadherin) on TC, than on 4 kPa substrates.

The detailed dynamics could be further resolved by subclustering and RNA velocity. The subclustering identified the stiffness-dependent intermediate state in Endo, Endo4, bridging Endo, Mes1, and Mes2 together. The Endo4 contained a higher fraction of the cells at the low stiffness conditions but not at the high stiffness. The difference suggested that Endo4 subcluster could be a critical transition state that drove the accumulation of the cells in mesenchymal-like clusters. The RNA velocity provided further details regarding the state transition based on the splicing dynamics of the RNA transcripts. Interestingly, the results revealed a distinct circulatory dynamic that present both EndMT and Mes-to-EndT transitions. The EndMT path was identified from Endo to Mes1 mainly through Endo1, the subcluster of Endo containing ECs at more activated state, and from Mes1 to Mes2, mainly through Mes2–1. The increase in Mes1–1 upon TGF-*β* induction suggested that TGF-*β* affected the conversion from Endo to Mes1. The Mes1–1 cluster also increased in the high stiffness conditions, but not as high as TGF-*β* induction, which could imply that stiffness also simultaneously promoted the transition from Mes1–1 to Mes2–1. The simultaneous increase in Mes2 along with the decreased fraction of Endo4 on high stiffness revealed that the stiffness-induced accumulation of Mes2 could be driven by inhibition of state conversion between Mes2 and Endo (the Mes2-to-EndT path). This was further confirmed by the trajectory analysis, showing minimal trajectory paths from Mes2 to Endo on high stiffness. Mes-to-EndT (reverse EndMT) or transient EndMT has been observed elsewhere, where ECs recover their naïve morphogenic and phenotypic characteristics, due to their cellular plasticity.^[4,38]^

The gene regulatory network analysis identified multiple critical correlations between transcription factors governing either EC or mesenchymal features. In the differential networks, the low stiffness condition did not present much difference compared to the common network, which suggested that no additional regulations governing the EC and mesenchymal features were activated. In contrast to the low stiffness network, the high stiffness network showed an increase in multiple edges within the common network involved additional EC specific transcription factors inhibiting the mesenchymal features, which demonstrated that the mesenchymal transition could be mediated by downregulation of these transcription factors at high stiffness.

Our analysis also successfully classified the ECs collected from human atherosclerotic lesions as Mes2, demonstrating that our in vitro system could closely mimic the pathological conditions associated with atherosclerosis. The classification of our in vitro samples to murine datasets combining both the normal ECs and atherosclerotic ECs revealed finer cell state details. The Endo and Mes1 subclusters with high endothelial features showed a higher classification score of normal ECs, while Mes2 subclusters were highly classified as atherosclerotic ECs. This finding again highlights the transcriptional similarity atherosclerotic ECs with Mes2, which was predominated in high stiffness or TGF-*β* induction. However, we fully understand that there might be discrepancies between in vitro culture and in vivo condition, and therefore, further in vivo studies are highly required to confirm whether stiffness-mediated substates and transcriptional feature of the modulates ECs are fully consistent with our in vitro model.

## 4. Conclusion

In conclusion, our studies at the transcriptome and protein level provide novel findings that high stiffness ECM promotes EC heterogeneity and phenotypic modulation. The scRNA-Seq revealed that the cell cluster with the highest endothelial features was enriched on substrates with physiological stiffness, whereas the clusters with mesenchymal transcriptional features were identified to be more populated on pathological substrates. The EC subsets induced by stiffness were enriched in human and murine atherosclerotic lesions, suggesting a new therapeutic approach to impede atherosclerosis progression through arterial stiffness. Furthermore, this study highlights the role of stiffness in atherosclerosis and serves as a census for the transcripts that drive the endothelial phenotypic changes at the single-cell level.

## 5. Experimental Section

### EC Culture and EndMT Induction:

Human coronary artery endothelial cells (ECs, passage 3–8, Lonza) were expanded in endothelial growth media (EGM-2MV, Lonza). Polyacrylamide hydrogel with stiffness of 4 or 100 kPa (Matrigen) were plated in 6-well dishes and coated with fibronectin (Sigma, 5 μg/cm^2^). Fibronectin-coated tissue culture plates (TC) were used as a control substrate that is of pathological stiffness. The amount of fibronectin on 4 kPa and TC was examined by immunofluorescence staining using a fibronectin-specific antibody (1:100, Invitrogen). For quantification, the surface fibronectin was dissociated using radioimmunoprecipitation assay (RIPA) buffer and the concentration was measured using Pierce Micro BCA Protein Assay kit (Thermo Scientific). The bioactivity of fibronectin on 4 kPa and TC substrates was also compared by culturing primary ECs on both substrates and then dissociating and counting the cells after 12 h. To study the effect of substrate stiffness, ECs were seeded at a density of 120 000 cells per well onto the hydrogels and TC. The day after cell seeding, the media was switched to a control medium consisting of endothelial basal medium (EBM, Lonza) supplemented with 5% fetal bovine serum (FBS, Lonza) but without exogenous growth factors. In some experiments, the control medium was further supplemented with TGF-*β* (20 ng mL^−1^, Peprotech) to induce EndMT. The medium was replaced daily over a course of 14 days.

### Immunofluorescence Staining and Analysis:

On day 14, cells were fixed in 4% paraformaldehyde (Alfa Aesar) for immunofluorescence staining, according to our previous publications.^[39,40]^ In brief, the cells were permeabilized in 0.5% Triton-X100 (Sigma) for 15 min. Cells were then blocked in 1% bovine serum albumin (Sigma) for 30 min. For primary antibody staining, cells were incubated with the antibody (VE-cadherin 1:40, R&D Systems; snail-1 1:100 Abcam; SM22*α* 1:100 R&D Systems; calponin 1:125 Abcam; Ki67 1:100, Sigma) overnight at 4 °C followed by Alexa Fluor-594 secondary antibody (Life Technologies). For staining of actin, cells were incubated with Alexa fluor-488-conjugated phalloidin (1:100, Life Technologies) for 2 h. Images were obtained using a Keyence BZ-X710 fluorescent microscope (*n* = 3). The immunofluorescence analysis was performed using ImageJ software. The quantification of snail-1 subcellular localization was carried out by subtracting the nuclear area (Hoechst staining) from total area of the snail expressing cells, followed by mean intensity measurements for cytoplasmic and nuclear area of the cells, and normalizing to cell counts. The measurements were repeated for three random 10× images obtained for each experiment and *n* = 3 independent experiments (total of 9 images per condition). For cell area measurements, 200 cells were manually measured in randomly selected areas of three images (10×) with phalloidin staining, and this was repeated for *n* = 3 independent experiments (total of 600 area measurements per condition). All the cell counts were carried out using the “Analyze particle” function in ImageJ, after appropriate thresholding. The SM22*α*, calponin and Ki67 positive cells were manually counted and corresponded to cell area measurements using “ROI manager” in ImageJ. The VE-cadherin intact junctional analysis was completed in FIJI. First, the mask that split the cells that consistently expressed VE-cadherin all the borders with ones that had a broken or diminished cell–cell junction was built. Subsequently, the number of nuclei that were covered by the mask by the built-in particle analysis function were quantified.

### Quantitative Real-Time Polymerase Chain Reaction:

Cell lysates were obtained at day 6 and 14 of culture, and the total RNA was isolated and purified using GeneJET RNA purification kit (ThermoFisher Scientific) according to the manufacturer’s instructions. The concentration of total RNA was measured using a UV-Vis spectrophotometer (NanoDrop 2000, Thermo Scientific), and the cDNA was synthesized from total RNA using the Superscript II reverse transcriptase kit (Life Technologies) following the manufacturer’s instructions, using a compact thermal cycler (T100 Thermal Cycler, Bio-Rad). Gene expression analysis was performed using quantitative real-time polymerase chain reaction (RT-PCR) of cDNA synthesized from purified RNA. RT-PCR was carried out using Taqman primers (Thermo Fisher Scientific) for CD31 (*PECAM1*), VE-cadherin (*CDH5*), TFPI2 (*TFPI2*), SM22*α* (*TAGLN*), calponin (*CNN1*), TPM2 (*TPM2*), MYL9 (*MYL9*), THY1 (*THY1*), snail-1 (*SNAI1*), and GAPDH using a 7900HT Sequence Detection System (Applied Biosystems). The data were quantified by the ΔΔCt method,^[41,42]^ normalized to GAPDH housekeeping gene, and then expressed as relative fold changes (*n* = 3).

### Single-Cell RNA Sequencing:

At day 14, cells cultured on 4 kPa hydrogel and TC with and without TGF-*β* stimulation were dissociated and resuspended in DMEM supplemented with 10% FBS. Single Cell libraries were prepared using a 10× Chromium v3 microfluidic chip, targeting 8000 cells per sample. The barcoded libraries from individual samples were then pooled and multiplexed, followed by sequencing on an Illumina NovaSeq 6000 platform using a NovaSeq-S1 (2 × 100 bp) flow cell. All the single cell capture, library preparation and sequencing were performed at Stanford Genome Sequencing Service Center.

### Single-Cell RNA Sequence Alignment:

The FASTQ files for each in vitro sample were generated from the raw bcl data by bcl2fastq. The FASTQ files were subsequently processed using Cell Ranger 3.0 with default parameters. In brief, the alignment was performed by STAR with the human reference genome GRCh38 to generate the bam file. The BAM files were then subjected to the Velocyto pipeline to estimate the unspliced and the spliced read counts. For the in vivo dataset, the raw unaligned data was obtained from the GEO database (GSE131780).

### Quality Control and Clustering:

For both in vitro and in vivo human dataset, the aligned scRNA-Seq data were processed on Scanpy platform.^[43]^ The low-quality cells that expressed less than 200 genes, or the expression of mitochondria genes exceeded 25% of total counts, were removed. Any genes that expressed less than three cells were excluded. In total, 21 644 high quality cells were obtained from the in vitro dataset. The gene counts were normalized to 10 000 per cell and the most variable genes were identified by the minimal dispersion of 0.5. Subsequently, cell cycle genes were regressed out before clustering to avoid interference caused by cell cycle phases. After clean-up, the individual cells were clustered under the unsupervised Leiden method. For visualization purposes, cells with UMAP coordinates were embedded. The differential genes for each cluster were evaluated by t-test. The in vivo dataset contained more complex cell structure, including smooth muscle cells, fibroblast, endothelial cells, and immune cells. To specifically compare the endothelial cells, all the clusters that do not belong to the endothelial clusters were excluded by identifying the most differential genes. For the in vivo murine dataset, we obtained the expression matrix from the GEO database (GSE150644), followed by data clean-up the same as above. Both samples collected from the atherosclerotic lesion and normal aorta was included, and subsequently excluded clusters do not belong to ECs.

### Marker Prediction:

The tool COMET^[[44]]^ was used to identify the most specific marker to represent each distinct cluster for RNAscope analysis. Three required input data frames were prepared, one represented the visualization coordinates, one contained the original expression matrix, and one indicated the Leiden clustering information. The three data frames were subjected to the web-based interface (http://www.cometsc.com/comet). The top specific markers were selected as the ones for the RNAscope analysis.

### Single-Cell Classification:

The classification for two independent samples was done by SingleCellNet^[45]^ (http://github.com/pcahan1/singleCellNet/) by a two-step protocol that consisted of classifier building, and query data classification. First, the classifier was built by the preprocessed scRNA-seq data with cluster annotation. For each cluster, the top ten most differentially expressed genes were identified and ranked the top 25 gene-pairs. Based on the selected gene pairs, the preprocessed training data were subsequently transformed and used to build a random forest classifier of 1000 trees. Second, the query data were classified by using the trained classifier to obtain the results. The cells that were not classified as any cell types of the reference data would be considered as miscellaneous.

### Trajectory Analysis:

To identify the transitions between cell states, RNA velocity was calculated by scVelo.^[46]^ The raw data were piped into Velocyto to generate a loom file with exon and intron annotation. Subsequently, scVelo was applied to obtain RNA velocity which was projected to either UMAP or diffusion map. In brief, the top 2000 most variable genes with a minimum of 20 expressed counts and 10 unspliced counts was identified. Subsequently, the nearest-neighbor graph was computed with 30 neighbors based on the Euclidean distances in space with 30 principal components. The first and second order moments were computed for each cell across its 30 nearest neighbors. The RNA velocity for each cell was then estimated. The cells were embedded on UMAP coordinates generated from the Scanpy analysis. PAGA was applied to evaluate the connectivity between each distinct cluster, using the function tl.paga in Scanpy. Approximately 10 nearest neighbors were used to compute the symmetrized kNN-like graph. PAGA connectivity was subsequently evaluated for each pair of clusters. To preserve the topology of the original coordinates, the nodes representing clusters were embedded on the median coordinates of the cluster.

### Gene Set Enrichment Analysis:

To identify the function of the top differential genes, we used GSEApy^[47–49]^ to perform the genesets enrichment analysis. The top 200 differential genes from each cluster were subjected to enrichr function. The most significant gene-sets that existed in three different databases were evaluated, which were Gene Ontology biology process, reactome and WikiPathway.

### Gene Regulatory Network Reconstruction:

To identify the key regulators governing the transcriptome, the context likelihood of relatedness (CLR) was used.^[50]^ In brief, the transcription factors were identified and correlation between genes were calculated. The mutual information matrix (mim) was built and subsequently piped the matrix into clr function from the package minet. The gene regulatory network (GRN) was reconstructed based on the correlation values. To compile the common network across different conditions, a stringent criterion was applied that only identifies the transcription factor-target pairs that exist across all four samples. To identify the stiffness-specific network, the unique transcription factor-target pairs were identified that only belong to each sample, and again found the common scription factor-target pairs that exist across the same stiffness. To identify the cluster-specific networks and evaluate the correlation between these networks and mechanotransduction elements, the networks using the cluster subset was reconstructed and included the edges that have direct connectivity to the nodes belongs to mechanotransduction.^[29]^

### Fluorescent In Situ Hybridization:

Fluorescent in situ hybridization was performed using Advanced Cell Diagnostics RNAscope probes and reagents. HCAECs cultured for 14 days on TC with TGF-*β* stimulation were fixed in 10% neutral buffered formalin for 30 min at RT, and then dehydrated using series of ethanol solution with increasing concentration (i.e., 50%, 70%, and 100%) and stored at −20°C. For hybridization of the probes, first the cells were hydrated again using series of ethanol solutions with decreasing concentrations, then endogenous peroxidase activity was quenched with hydrogen peroxide reagent for 10 min, followed by protease digestion for 10 min at RT. Then the target probes and positive probes were hybridized for 2 h at 40°C in a humidity-controlled oven (HybEZ II, ACDbio). The target probes that were used were as follows: Hs-TFPI2-C1, Hs-PXDN-C2, Hs-TAGLN-C3, and Hs-CD44-C4. Subsequently, a series of amplifications were carried out for each channel using the proprietary AMP reagents, and then the fluorescent signal was developed using probe-specific HRP-based amplification and Opal dyes conjugation (Opal 520, Opal 570, Opal 620, or Opal 690; Akoya). Cells were then stained for nuclei with DAPI, and coverslipped with Prolong Gold antifade mounting medium and stored at 4 °C. Image acquisition was carried out using a Zeiss laser scanning confocal microscope (LSM880). For analysis of the spatial distribution of different cell populations in RNAscope images, R on the “Enable Cloud Workbench” software was used and explored the distances between cells and their immediate neighbors by computing a k-nearest neighbor graph on the cell coordinates with k = 5 (“kNN” function from the package “dbscan”) and measuring the distance between each cell and its neighbors. The average cell-cell distance between groups of cells was also computed, regardless of their graph structure, by using the “rdist” function from the “rdist” package. To visualize the spread of cell types across a sample, 2D kernel density estimates from cell coordinates was generated using the “stat_density_2d” function from the “ggplot2” package with “bins = 5”. To compute the area of this spread, the 75% density contour was extracted as coordinate points (“ggplot_build”) and converted to “SpatialPolygons” objects using the “sp” package. The 2D area of the contours was pulled from these objects and summarized across RNAscope experiments (n = 3). The Fiji plugin “Spatial Statistics 2D/3D” was used also to quantify the spatial distribution of the cells in the images. The G-function which is defined based on the distance between a typical point in the pattern and its nearest-neighbor was calculated for 3 images from 3 different experiments and the result was presented as mean ± STD.

### Statistical Analysis

Statistical analysis was performed using GraphPad Prism 9. For multiple group comparison, one-way ANOVA was performed followed by Tukey’s multiple comparisons test with a significance threshold of *p* < 0.05. For comparison of two groups of data, unpaired student’s *t*-test was performed with a significance threshold of *p* < 0.05. The data were presented as mean ± STD.

## Supplementary Material

supinfo

## Data Availability

The data that support the findings of this study are available from the corresponding author upon reasonable request.
