# Supplementary material for: Single-Cell Transcriptomic Census of Endothelial Changes Induced by Matrix Stiffness and the Association with Atherosclerosis
Source: Adv Funct Mater. Author manuscript; Available in PMC 2023 Nov 17. (PMC9937733; doi:10.1002/adfm.202203069)
Supplement: supinfo [file NIHMS1855958-supplement-supinfo.pdf]

ADVANCED FUNCTIONAL MATERIALS

Supporting Information

for *Adv. Funct. Mater.,* DOI: 10.1002/adfm.202203069

Single-Cell Transcriptomic Census of Endothelial Changes Induced by Matrix Stiffness and the Association with Atherosclerosis

Maedeh Zamani, Yu-Hao Cheng, Frank Charbonier, Vivek Kumar Gupta, Aaron T. Mayer, Alexandro E. Trevino, Thomas Quertermous, Ovijit Chaudhuri, Patrick Cahan, and Ngan F. Huang**

Supplementary Materials for

Single-Cell Transcriptomic Census of Endothelial Changes Induced by Matrix Stiffness and the Association with Atherosclerosis

Maedeh Zamani, Yu-Hao Cheng, Frank Charbonier, Vivek Kumar Gupta, Aaron T. Mayer, Alexandro E. Trevino, Thomas Quertermous, Ovijit Chaudhuri, Patrick Cahan, Ngan F. Huang**

*Corresponding author. Email: ngantina@stanford.edu; patrick.cahan@jhmi.edu

This PDF file includes:

Figure. S1 to S11
Table S1

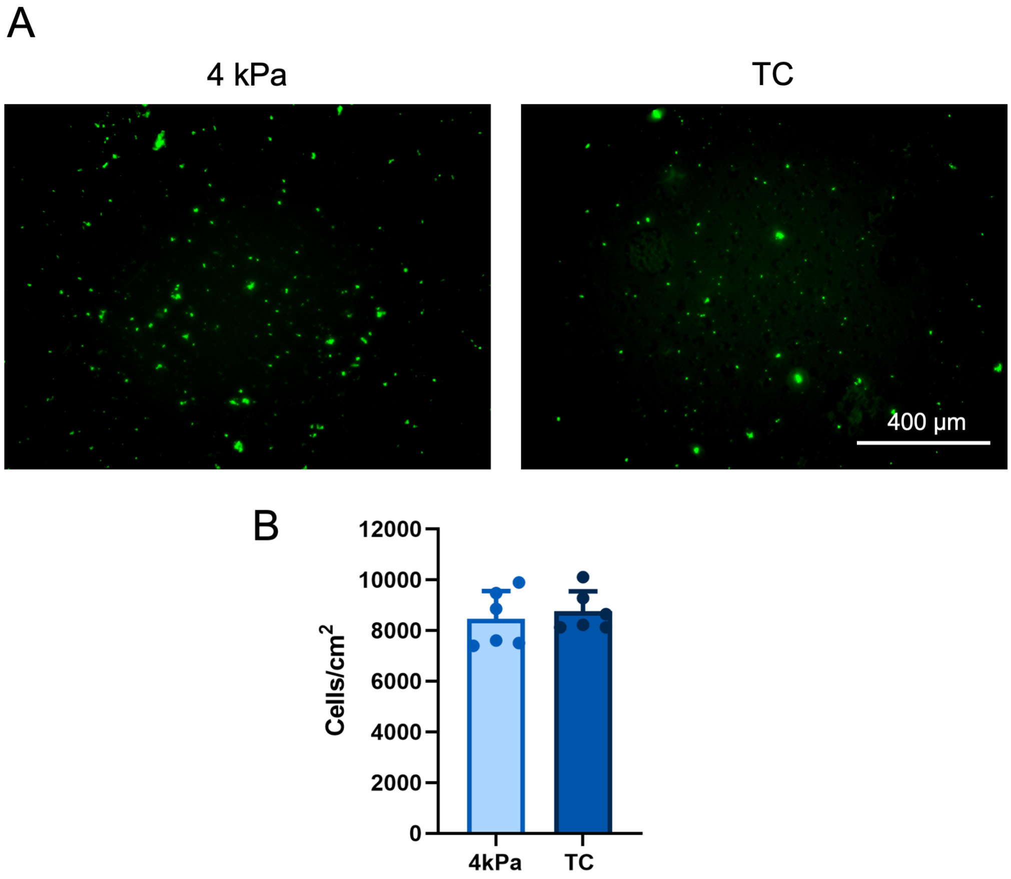

Figure S1. Characterization of fibronectin bioactivity. (A) Immunofluorescence staining of fibronectin on 4 kPa and TC substrates. Images were acquired using 10X objectives and the scale bar represents 400 μm. **(B)** Quantification of the initial cell attachment to 4 kPa and TC substrates modified with fibronectin. Data shown as mean ± STD and data points represent each sample (n=6).

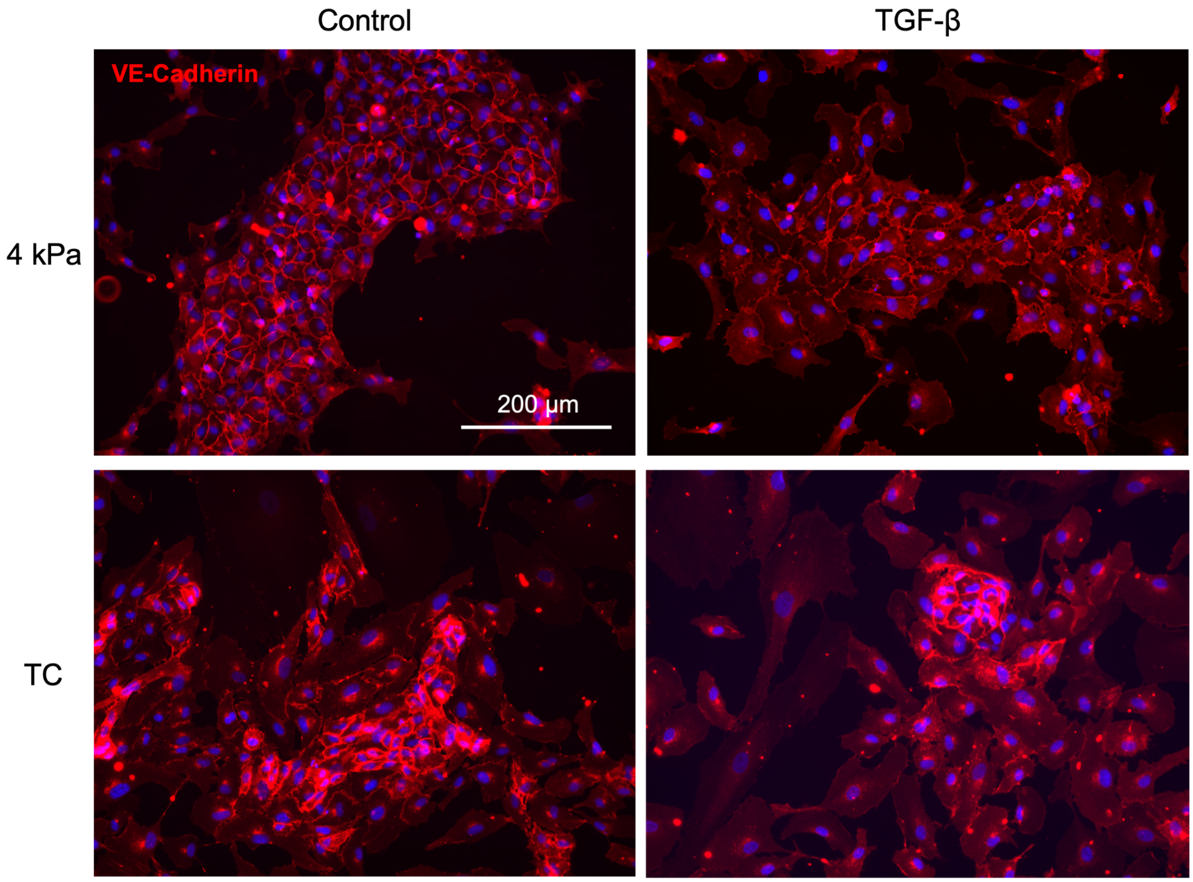

Figure S2. Substrate stiffness induced morphological changes in ECs. Immunofluorescence staining of VE-cadherin expression of HCAECs cultured on 4 kPa and TC substrates, without (Control) and with TGF-β stimulation after 14 days. Images were acquired at 20X and the scale bar represents 200 µm.

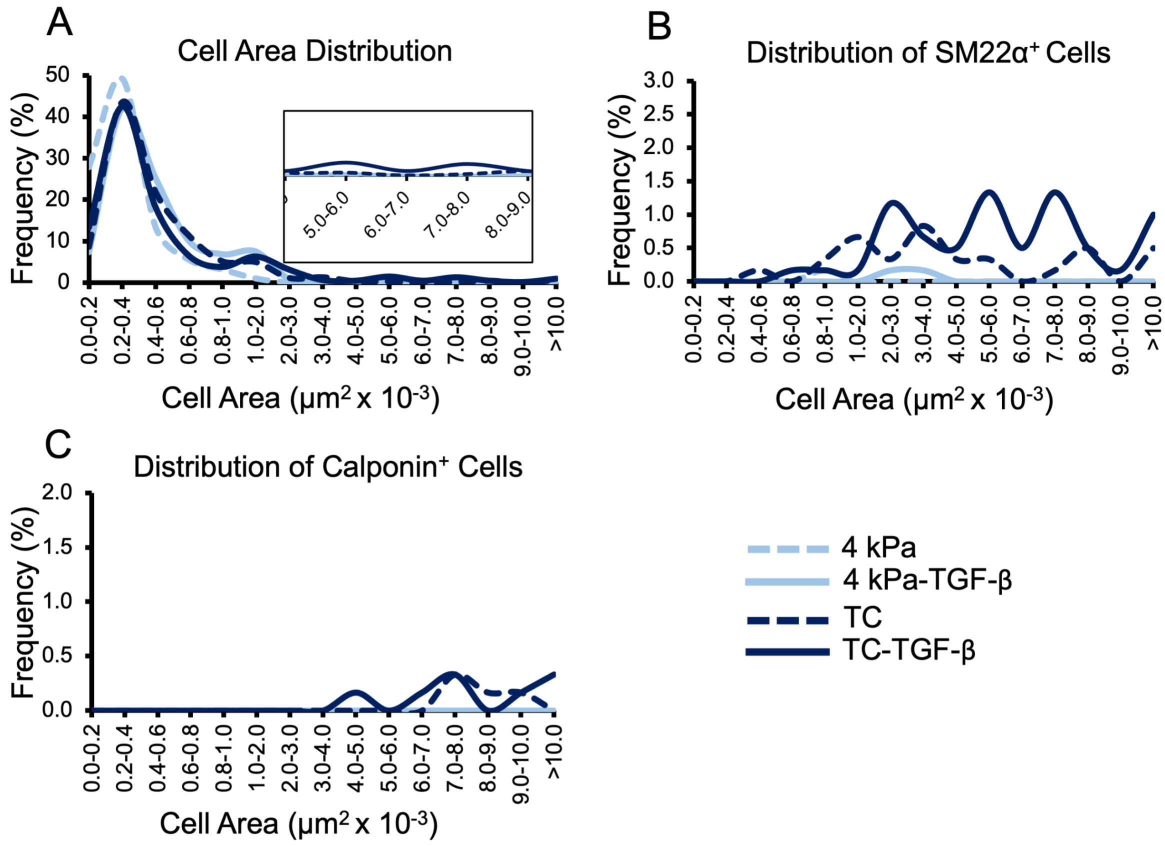

Figure S3. Substrate stiffness induced morphological heterogeneity in ECs. (**A**) Histogram of the cell area distribution revealed a fraction of the cells with higher cell spreading on TC. Histogram of (**B**) SM22α and (**C**) Calponin expressing cells illustrated the association of these mesenchymal markers with cell sizes larger than 1000 µm^2 that were more frequent on TC (*n*=3).

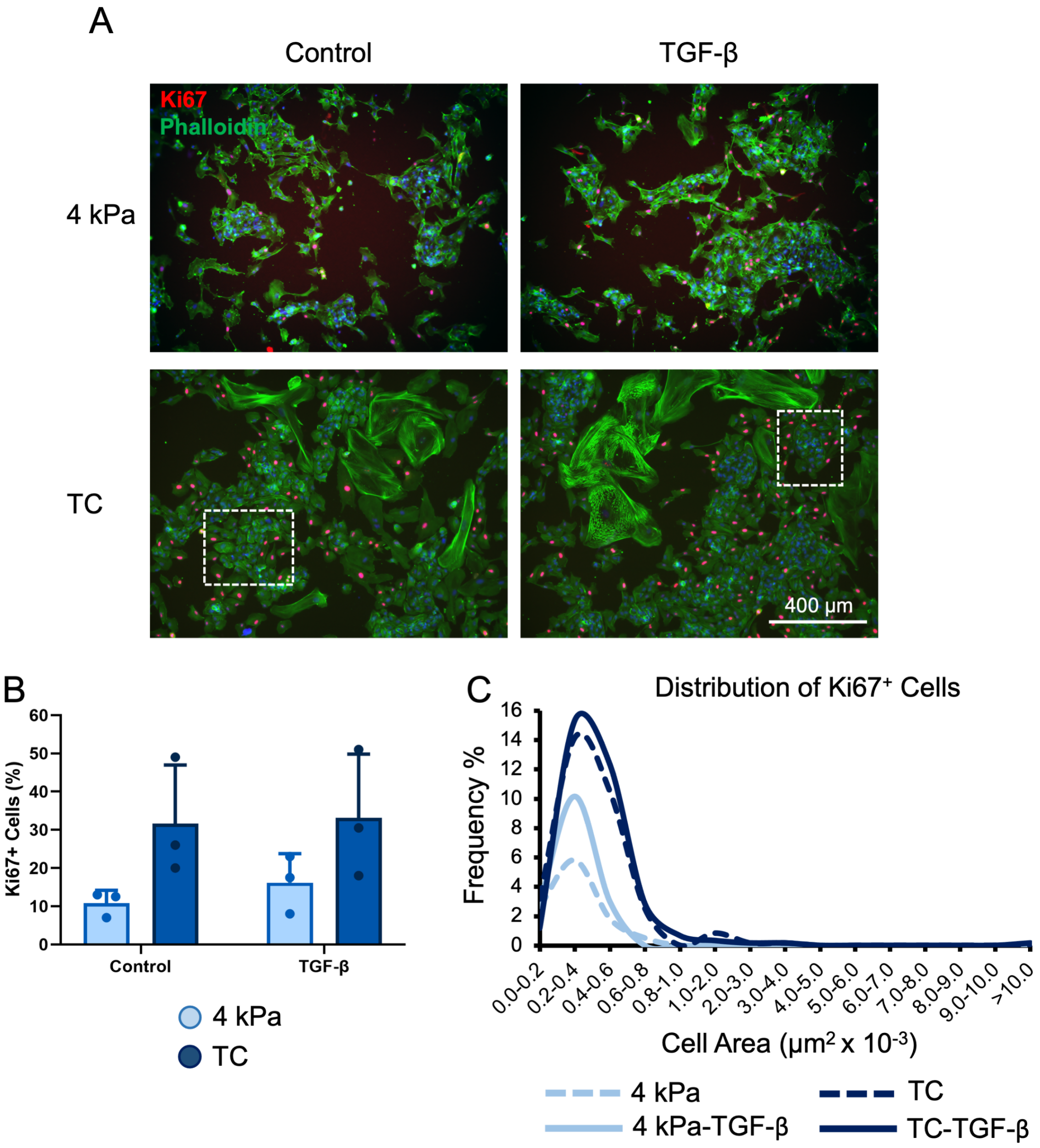

Figure S4. Transitioning ECs were in the proliferative state. (**A**) Immunofluorescence staining of Ki67 and F-actin (phalloidin) for human coronary artery ECs cultured on 4 kPa substrate and TC, without (Control) and with TGF-β stimulation after 14 days. Transitioning cells that appeared detached from the multi-cellular clusters stained positive for Ki67 and were in the proliferative state. (**B**) Quantitative analysis of Ki67-positive cells on substrates of varying stiffness without (Control) and with TGF-β stimulation. (**C**) Histogram of $Ki67^+$ cells demonstrated that the proliferative cells had a cell area of <1000μm^2/cell or relatively larger area of 1100-4000 μm^2/cell. Scale bar represents 400 μm. Data are shown as mean ± STD (*n*=3). Data points represent average for each *n*.

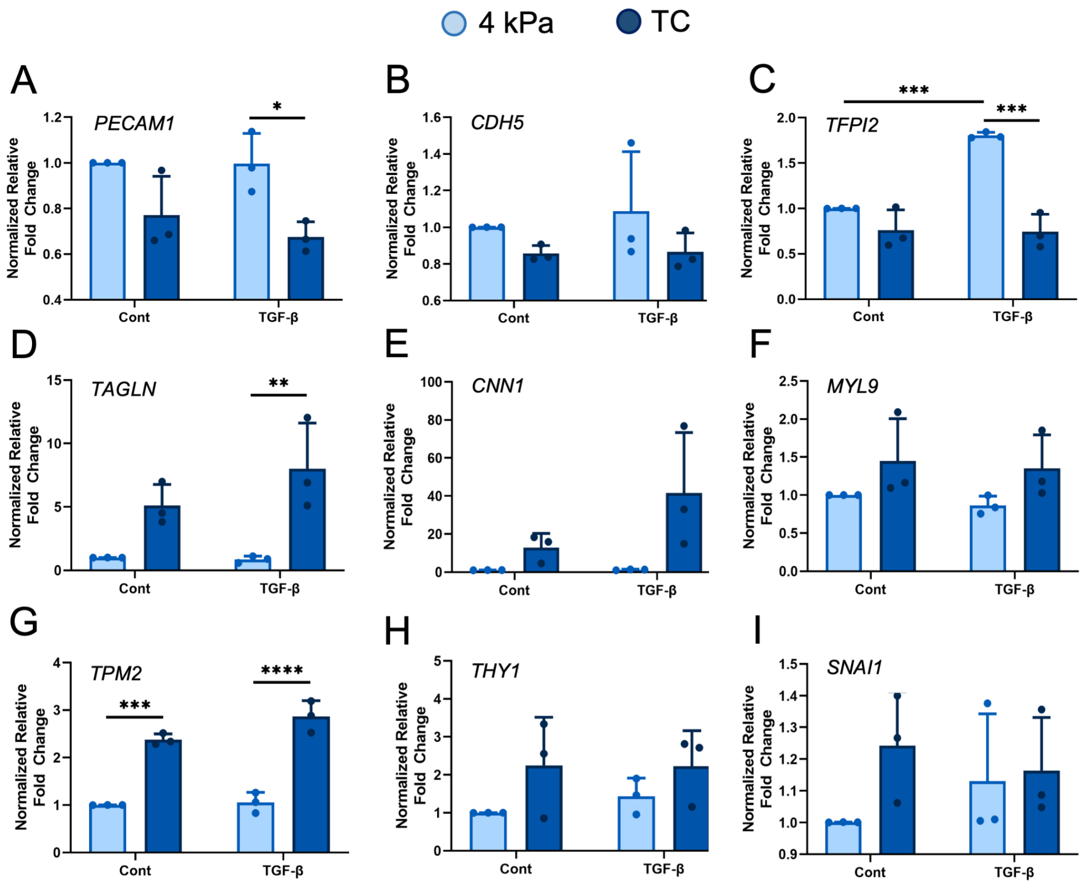

Figure S5. EC phenotypic modulation induced by matrix stiffness is validated by qPCR. Quantitative gene expression analysis of stiffness-induced ECs phenotypic modulation. Relative gene expression of (**A**) *PECAM1* (**B**) *CDH5* (**C**) *TFPI2* (**D**) *TAGLN* (**E**) *CNN1* (**F**) *MYL9* (**G**) *TPM2* (**H**) *THY1* (**I**) *SNAI1* for human coronary artery ECs cultured on 4 kPa substrate and TC, without (Control) and with TGF-β stimulation after 6 days. Gene expressions are normalized to GAPDH and are relative to 4 kPa as control. Data are shown as mean ± STD (*n*=3). Data points represent average for each *n*. *p < 0.05, **p < 0.005, ***p < 0.0005, ****p < 0.00005.

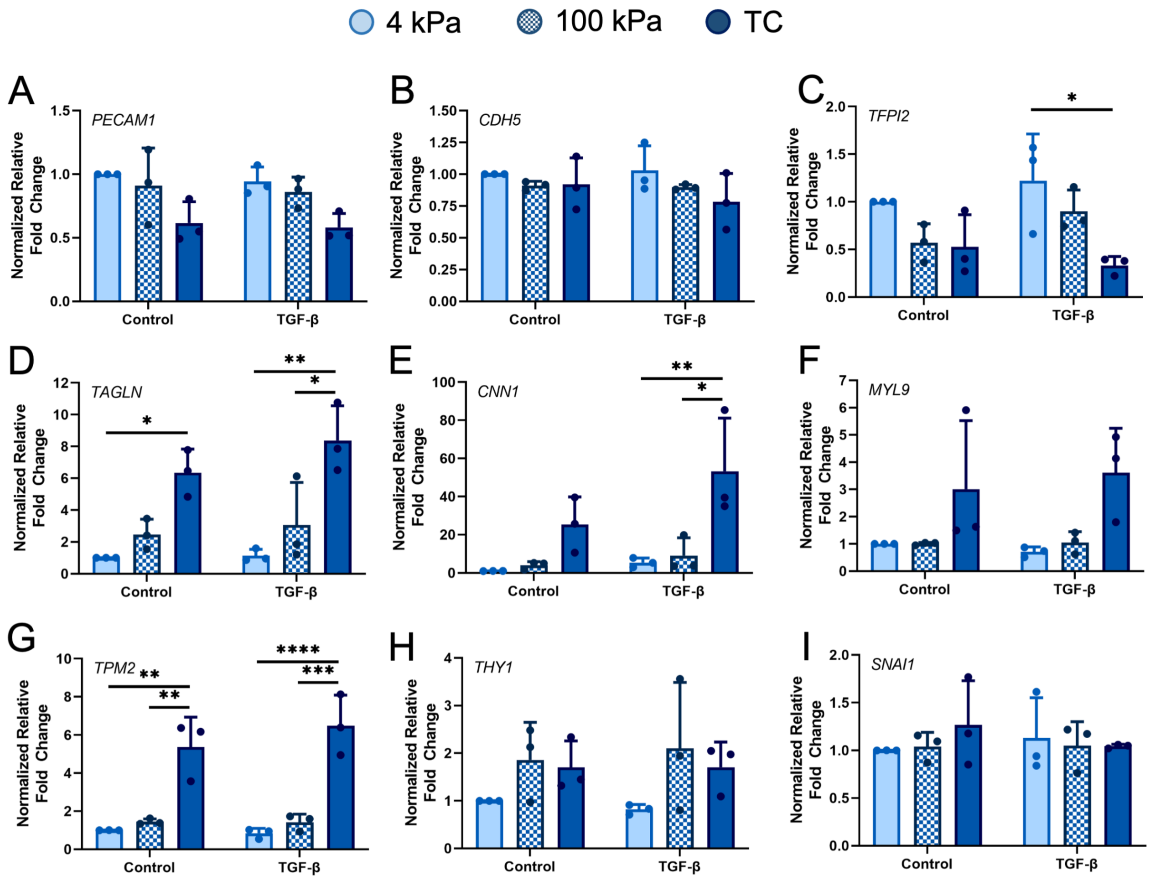

Figure S6. EC phenotypic modulation induced by matrix stiffness is validated by qPCR. Quantitative gene expression analysis of HCAECs cultured on 4 kPa, 100 kPa and TC, without (Control) and with TGF-β stimulation after 14 days. Relative gene expression of (**A**) *PECAM1* (**B**) *CDH5* (**C**) *TFPI2* (**D**) *TAGLN* (**E**) *CNN1* (**F**) *MYL9* (**G**) *TPM2* (**H**) *THY1* and (**I**) *SNAI1*. Gene expressions are normalized to *GAPDH* and are relative to 4 kPa as control. Data are shown as mean ± STD (n=3). Data points represent average for each n. *$p < 0.05$, **$p < 0.005$, ***$p < 0.0005$, ****$p < 0.00005$.

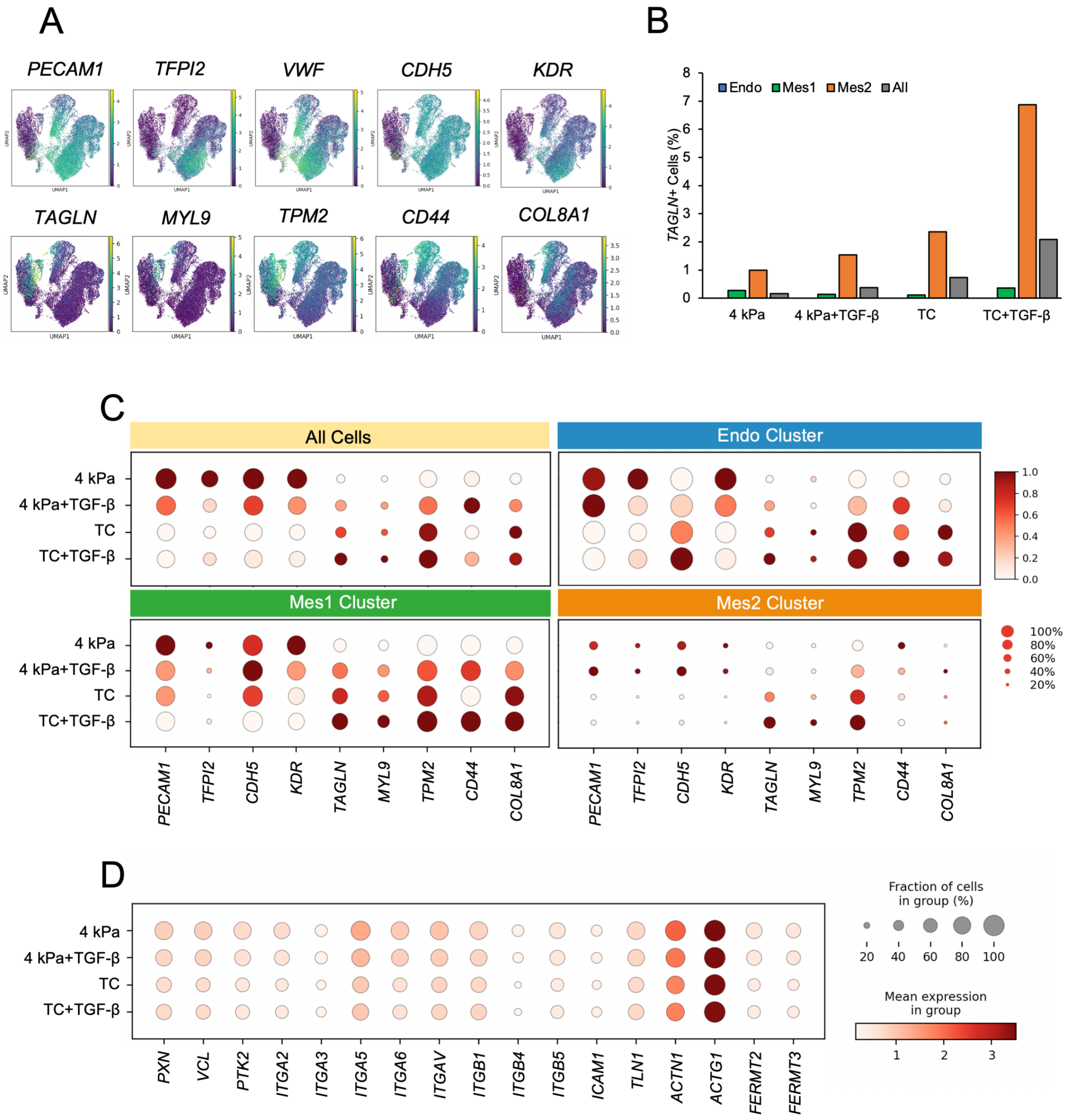

Figure S7. Three identified clusters were transcriptionally distinct from each other. (**A**) The expression level of selected EC and mesenchymal genes in UMAP. (**B**) The distribution of *TAGLN*+ cells across three clusters. The number of *TAGLN*+ cells increased on stiff matrix or upon TGF-β induction. (**C**) The pseudo-bulk comparison across culture conditions revealed endothelial features, such as *PECAM1*, *TFPI2* and *KDR*, were downregulated at high stiffness and TGF-β stimulation at total cell population and individual cluster level, while mesenchymal features, including *TAGLN*, *MYL9* and *TPM2* were upregulated. **(D)** The gene expression level of integrins, adhesion molecules and cytoskeletal related genes was consistent across four different culturing conditions.

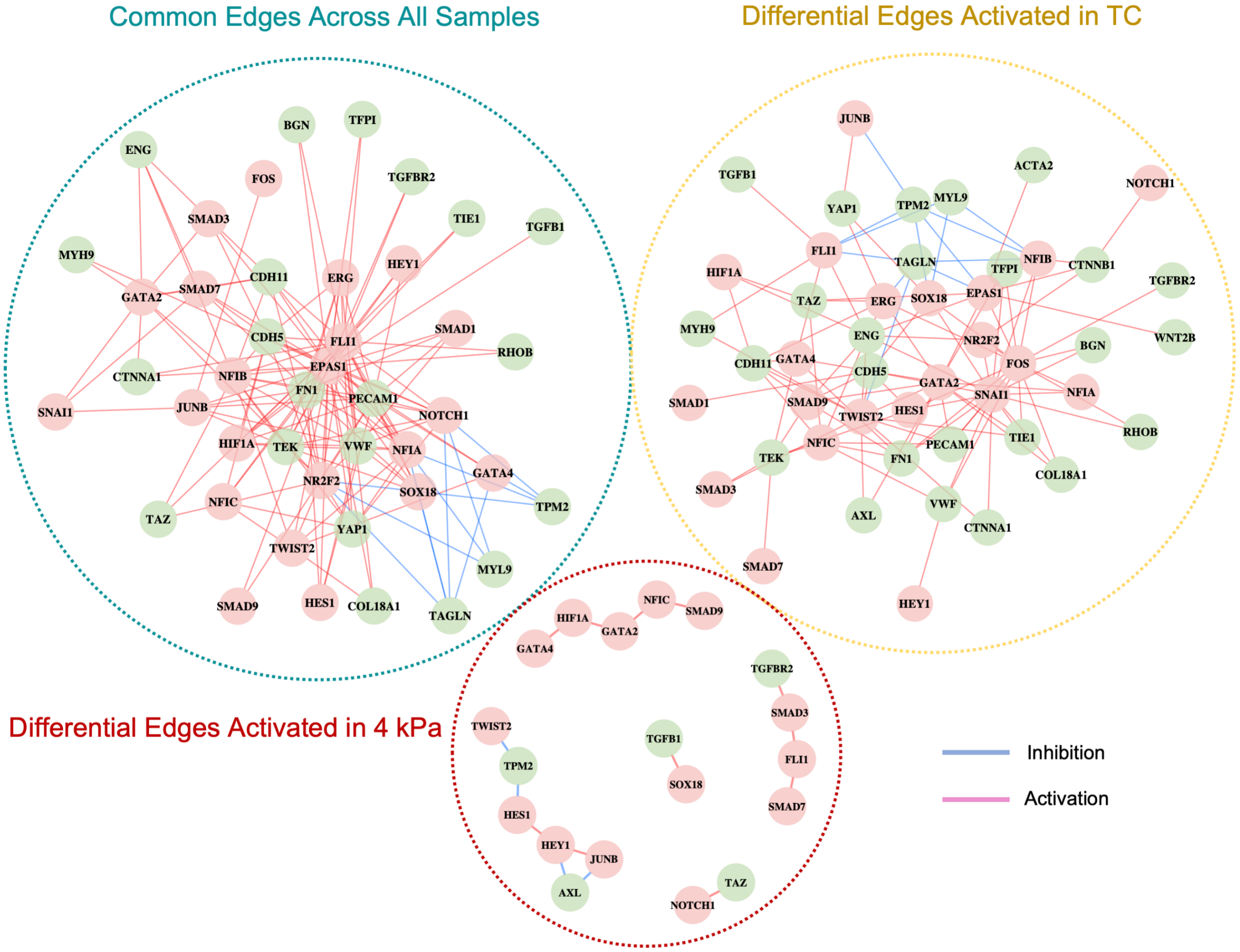

Figure S8. Gene regulatory networks identified common and specific stiffness regulated transcription factor-target gene edges. A shared network involving multiple transcription factors that were correlated to EC features such as *CDH5*, *VWF*, *PECAM1*, and mesenchymal features such as *TPM2*, *TAGLN* and *MYL9* were identified across different culture conditions by context likelihood of relatedness (CLR). The differential analysis revealed a significant increase in network complexity at high stiffness conditions by regulating multiple edges between the transcription factors and targets.

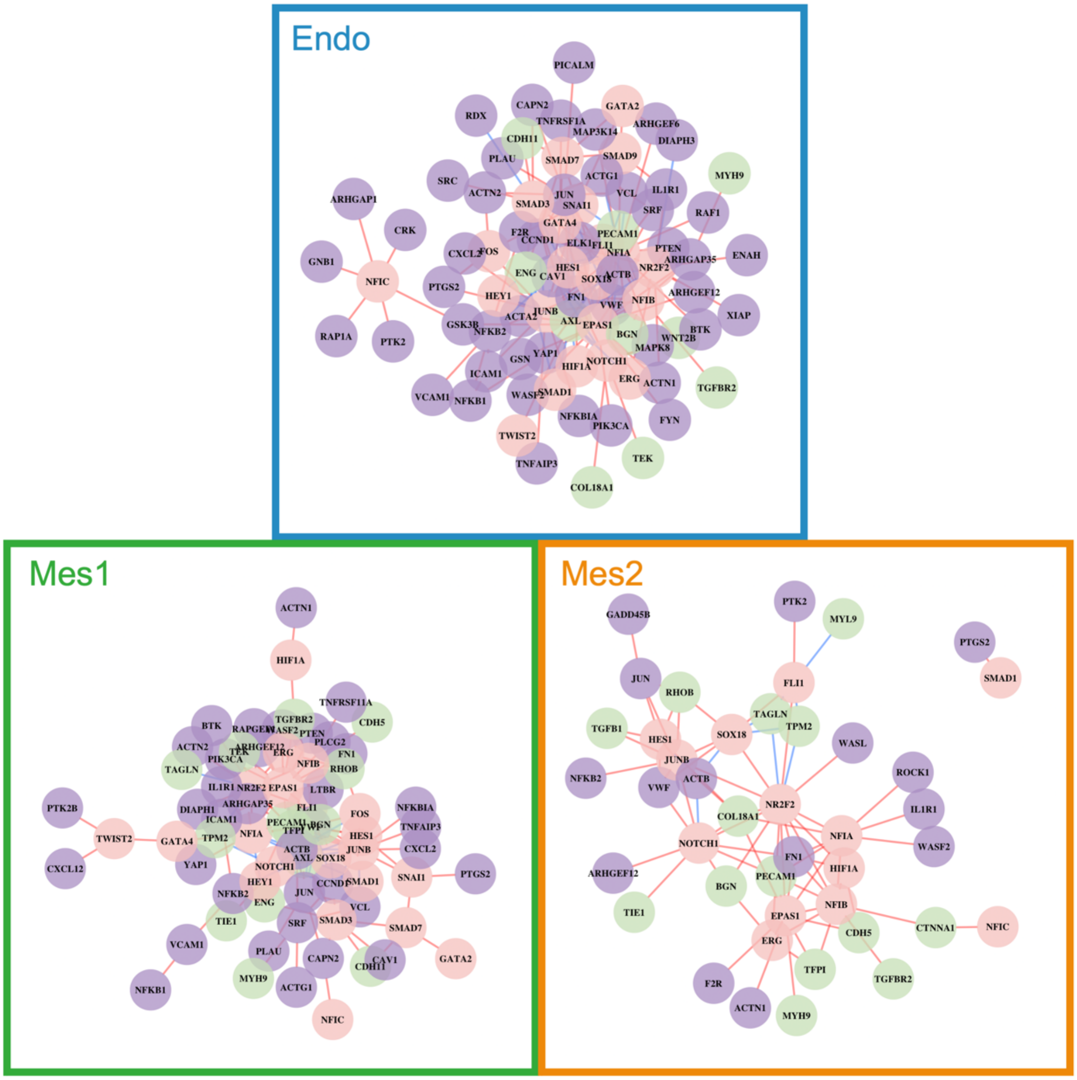

Transcription factor | Target gene | Mechanotransduction element

Figure S9. Gene regulatory networks identified the correlation between cluster-specific transcription factor-target gene edges and mechanotransduction elements. The cluster-specific gene regulatory networks revealed a high degree of connectivity between endothelial and mesenchymal features and their regulators to the mechanotransduction elements in Endo, which was gradually decreased by transition to Mes1 and Mes2.

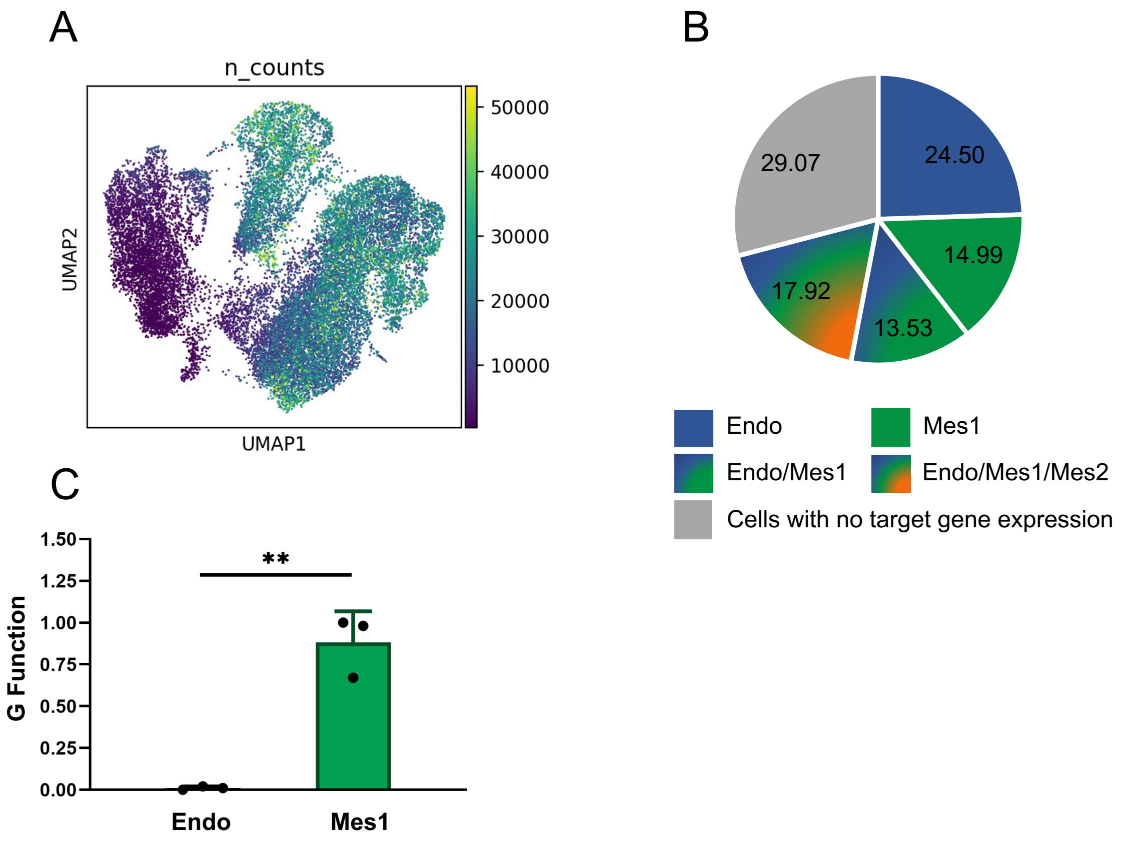

Figure S10. (**A**) The UMAP of gene counts for single cells across three clusters. A global gene silencing was observed for the cells in Mes2. (**B**) Pie chart illustrates the Percentage of the cells in each cluster identified by RNAscope probes (*n*=3). (**C**) Quantification of spatial distribution of Endo and Mes1 cells using G-function. G values confirmed the clustered distribution of Endo cells and random distribution of Mes1 cells in the culture. Data are shown as mean ± STD (*n*=3). Data points represent average for each *n*. $^{**}p < 0.005$.

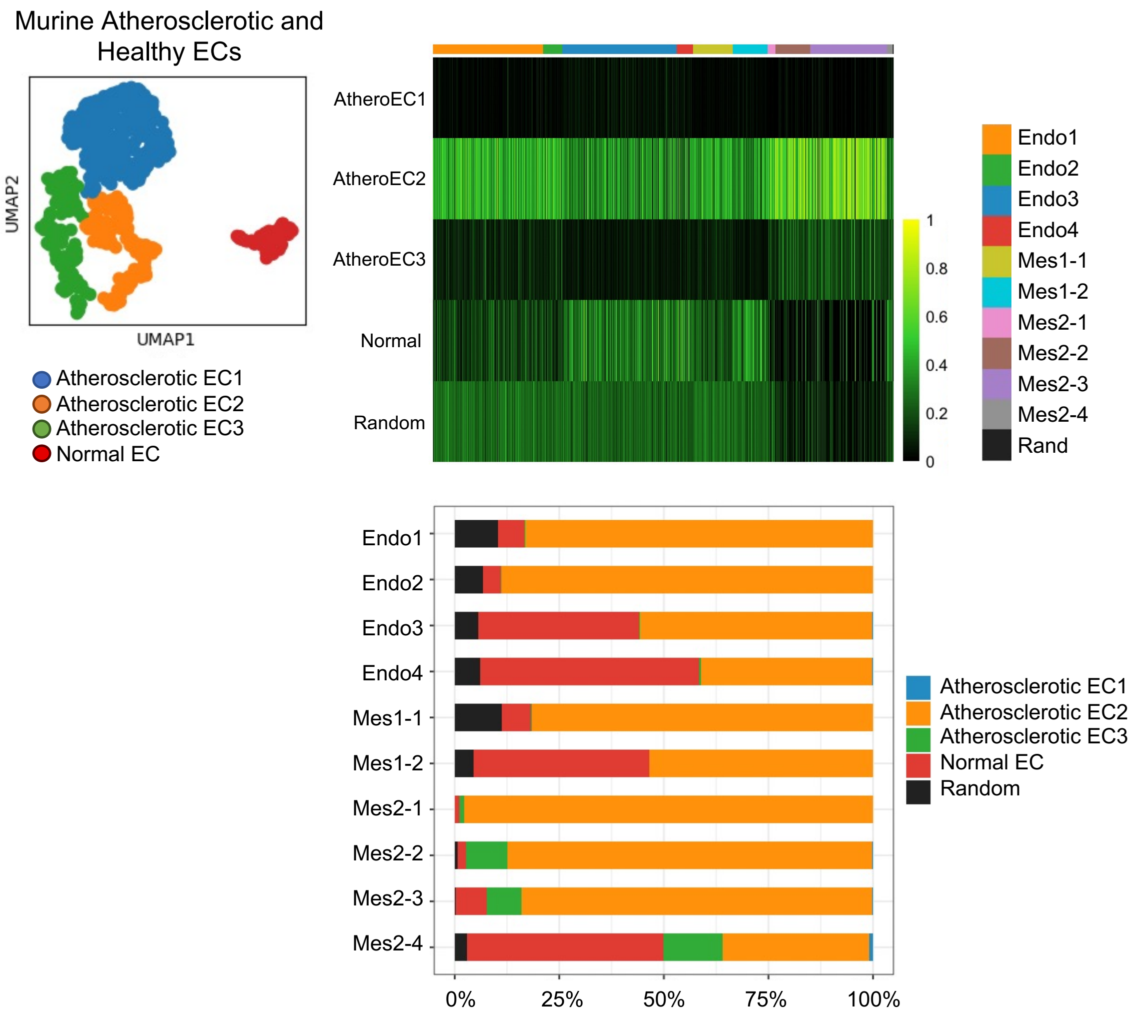

Figure S11. Phenotypically modulated human ECs from *in vitro* culture were transcriptionally similar to murine atherosclerotic ECs. Single-cell classification of our in vitro cultured human ECs on murine healthy and atherosclerotic ECs. The results revealed Endo3, Endo4, and Mes1-2 to be partially classified as healthy ECs, while the rest of the subclusters were highly classified as murine atherosclerotic ECs.

Gene Name
TAGLN
APOE
TPM1
TPM3
POSTN
COL1A2
MYL9
ABCA1
NEXN
S100A11
CCL2
MYO5A
DDR2
PLEK2
NT5E
VIM
PRKCA
FBLN5
CTGF
PTX3

Table S1. Selected features identified from human atherosclerotic lesions. Compiled list of genes that included features identified in human atherosclerotic sequencing data and relevant to EndMT to build a reference geneset.
